# Type-I Prenyl Protease Function Is Required in the Male Germline of *Drosophila melanogaster*

**DOI:** 10.1534/g3.112.002188

**Published:** 2012-06-01

**Authors:** Katie Adolphsen, Amanda Amell, Nathan Havko, Sara Kevorkian, Kyle Mears, Hayley Neher, Dietmar Schwarz, Sandra R. Schulze

**Affiliations:** Biology Department, Western Washington University, Bellingham, Washington 98225

**Keywords:** prenylation, human disease, fertility, molecular evolution, mutagenesis

## Abstract

Many proteins require the addition of a hydrophobic prenyl anchor (prenylation) for proper trafficking and localization in the cell. Prenyl proteases play critical roles in modifying proteins for membrane anchorage. The type I prenyl protease has a defined function in yeast (Ste24p/Afc1p) where it modifies a mating pheromone, and in humans (Zmpste24) where it has been implicated in a disease of premature aging. Despite these apparently very different biological processes, the type I prenyl protease gene is highly conserved, encoded by a single gene in a wide range of animal and plant groups. A notable exception is *Drosophila melanogaster*, where the gene encoding the type I prenyl protease has undergone an unprecedented series of duplications in the genome, resulting in five distinct paralogs, three of which are organized in a tandem array, and demonstrate high conservation, particularly in the vicinity of the active site of the enzyme. We have undertaken targeted deletion to remove the three tandem paralogs from the genome. The result is a male fertility defect, manifesting late in spermatogenesis. Our results also show that the ancestral type I prenyl protease gene in *Drosophila* is under strong purifying selection, while the more recent replicates are evolving rapidly. Our rescue data support a role for the rapidly evolving tandem paralogs in the male germline. We propose that potential targets for the male-specific type I prenyl proteases include proteins involved in the very dramatic cytoskeletal remodeling events required for spermatid maturation.

Prenylation is a posttranslational modification required by many different kinds of proteins to ensure their proper trafficking and localization. Prenylated proteins have in common a four amino acid C-terminal motif called a CaaX box (C = cysteine, a = aliphatic, and X= a wide but not unlimited range of residues). Prenylation involves the addition of a hydrophobic polyisoprenoid group to the CaaX-box cysteine, proteolytic cleavage of the terminal three amino acids (-aaX), and carboxymethylation of the now exposed cysteine hydroxyl group [reviewed in Nguyen *et al.* (2010)]. In addition, vertebrate A-type lamin proteins and yeast (*Saccharomyces cerevisiae*) a-factor mating pheromone undergoes further proteolytic cleavage on the N-terminal side of the CaaX motif, releasing a prenylated peptide. Yeast mating pheromones and A-type lamins share nothing in common other than this rather elaborate processing. In yeast, the cleaved prenylated peptide serves a downstream signaling function (Chen *et al.* 1997; Boyartchuk and Rine 1998). However, in vertebrates, it is not yet clear why A-type lamins exhibit only transient prenylation. The paralogous B type lamins maintain their prenylated anchor, suggesting a housekeeping supportive role in the lamina underlying the nuclear envelope (Capell and Collins 2006). It is possible that transient prenyl processing is required for A-type lamins so they can be released into the nuclear interior to regulate processes such as replication and gene expression (Dechat *et al.* 2010). Interestingly, a mouse genetically engineered to only express a CaaX-less splice alternative of the A-type lamin gene product called Lamin C is perfectly viable, which suggests the alternative hypothesis that transient prenylation of A-type lamins is a mechanism to remove excess lamin from the lamina (Fong *et al.* 2006). Loss-of-function mutations in the human A-type lamin gene segregate with a wide range of predominantly tissue-specific but nonlethal diseases, supporting a role in regulating gene expression (Worman *et al.* 2010). A gain-of-function mutation in the A-type lamin that prevents the removal of the prenyl anchor causes a premature aging disorder called Hutchinson Gilford Progeria syndrome (HGPS) (Eriksson *et al.* 2003). Children with HGPS generally do not survive past adolescence, usually succumbing to a geriatric disease such as atherosclerosis (Pollex and Hegele 2004).

The enzyme in humans believed to be responsible for removing the prenyl anchor from the A-type lamin protein is the type I prenyl protease Zmpste24 (Barrowman and Michaelis 2009). This enzyme functions as a zinc metalloprotease and is highly conserved, and it was discovered by its ability to complement the homologous function in yeast (Tam *et al.* 1998). The yeast homolog, Ste24p (also known as Afc1p), overlaps in function with the type II prenyl protease Rce1p (Trueblood *et al.* 2000), and these two proteins may be responsible for most if not all proteolytic cleavage associated with prenyl processing (Boyartchuk *et al.* 1997; Krishnankutty *et al.* 2009). Genes encoding type I and type II prenyl proteases have been analyzed in *C. elegans* (Cadiñanos *et al.* 2003a), *A. thaliana* (Bracha *et al.* 2002; Cadiñanos *et al.* 2003b), mammals (Freije *et al.* 1999), and protozoans (Gillespie *et al.* 2007). In all of these organisms, single genes have been identified for each prenyl protease type. Typically, Rce1p is considered the major prenyl protease due to its well-studied role in processing small G-proteins critical for cell survival (Kim *et al.* 1999). Targets for the type I prenyl protease Ste24p include the yeast a-factor mating type pheromone and mammalian A-type lamin (Chen *et al.* 1997; Boyartchuk and Rine 1998; Corrigan *et al.* 2005), but additional targets are suggested because homozygous mutations in human *Zmpste24* cause a perinatal lethal disease called restrictive dermopathy, with phenotypic symptoms strongly resembling characteristics of HGPS (Navarro *et al.* 2005). A recent finding in *Drosophila* suggests the type I prenyl protease might also recognize a target serving as an attractant for germ-cell migration in the earliest stages of embryogenesis (Ricardo and Lehmann 2009). However, *Drosophila* is otherwise conspicuously absent from a survey of functional studies of type I prenyl proteases, likely due to the fact that the *STE24* gene has undergone at least two tandem duplications on the second chromosome in this species. (Yeast nomenclature is used throughout this article for general references to type I prenyl protease function). These tandem duplications have created three paralogs called *CG9000* (*ste24a*), *CG9001* (*ste24b*), and *CG9002* (*ste24c*); this arrangement is conserved throughout all Drosophilids so far fully sequenced (Drosophila 12 Genomes Consortium 2007) but is absent in all other sequenced insect genomes. Indeed, triplication of the type I prenyl protease appears to be a unique occurrence in all taxa based on current annotated genome sequence data. If one assumes biological redundancy in addition to shared functions with the type II prenyl protease [encoded in *D. melanogaster* by the single gene *CG4852* (*severas*)], it is perhaps not surprising why *Drosophila* type I prenyl protease activity should remain hidden from conventional mutagenesis screens (Castrillon *et al.* 1993; Wakimoto *et al.* 2004)

To determine the biological function of the type I prenyl protease in *Drosophila*, we have used homologous recombination to generate a triple knockout of the three tandem *STE24* paralogs. Our data show that a triple knockout has no effect on females but has a modest effect on male life span, in addition to almost complete male sterility. In addition, we find that the three paralogs are diverging in function. *CG9000* is the most highly conserved paralog based on amino acid sequence homology. The two additional paralogs, *CG9001* and *CG9002*, appear to have acquired a specific role in the male germline. We also find evidence via an evolutionary analysis for additional *STE24* paralogs in the *Drosophila* genome. *CG30461* is a coding region forming a dicistronic unit with *CG9002*, and it appears to be a highly diverged and truncated nonprocessed pseudogene. *CG7573* is a complete paralog located on the third chromosome, possibly arising due to retrotransposition. Both *CG30461* and *CG7573* show dramatic sequence changes, particularly in the critical regions of the protein containing the enzyme’s active site. In addition to completing a gap in the literature describing type I prenyl protease function in model organisms, our triple knockout lines provide valuable resources for further research on the biological process and consequence of prenylation.

## Materials and Methods

### *Drosophila* stocks and culture conditions

All of the fly stocks used for this study were obtained from Bloomington or Kyoto *Drosophila* stock centers or colleagues, or they were constructed for specific experiments. There are two independent PiggyBac elements in the promoter region of *CG9000* (*PBac{PB}ste24ac02930* and *PBac{RB}ste24ae00442*) and one in *CG9002/CG30461* (*PBac{PB}ste24c[c04444]*). All three are viable and fertile. Additionally, there is a *P* element mutation in the promoter of *CG9000* (*y[1] w[67c23]*; *P{w[+mC]=GSV6}GS14703*) that is also viable and fertile (although it possesses a second site female sterile mutation). All stocks were maintained on standard cornmeal agar medium. For fertility tests, newly eclosed virgin males were always used, and virgin females were never older than two days. All crosses were maintained at room temperature (20–22°). As noted in *Results*, the genomic region containing the *CG9000* cluster recombines readily with balancers. Stocks consisting of triple knockouts and Don Juan–GFP were constructed over *apterous^Xa^*, a reciprocal translocation between the second and third chromosomes, which placed the second chromosome *CG9000* region on the third chromosome, minimizing recombinatorial breakdown. For the rescue experiments, stocks could not be built over the *apterous^Xa^* reciprocal translocation as many of the transgenes were lethal when overexpressed and thus precluded homozygosity for the triple knockout deletion. Therefore, rescue stocks were built over *CyO;TM6*, enhancing balancer breakdown as described (see text). It was necessary to perform the heat shock in a dry incubator (monitored by a mercury thermometer) due to the large number of crosses that were performed simultaneously (approximately 200 individually tested males). Heat shocks were 37–38° for approximately 60 min daily from day 6 to day 8 after egg lay and throughout adulthood, and they were stopped only when no more progeny were evident. Constructed rescue stocks were discarded after data were collected (due to instability) and were constructed anew for each experiment.

### Molecular biology

#### Targeting construct:

**Figure 1:**
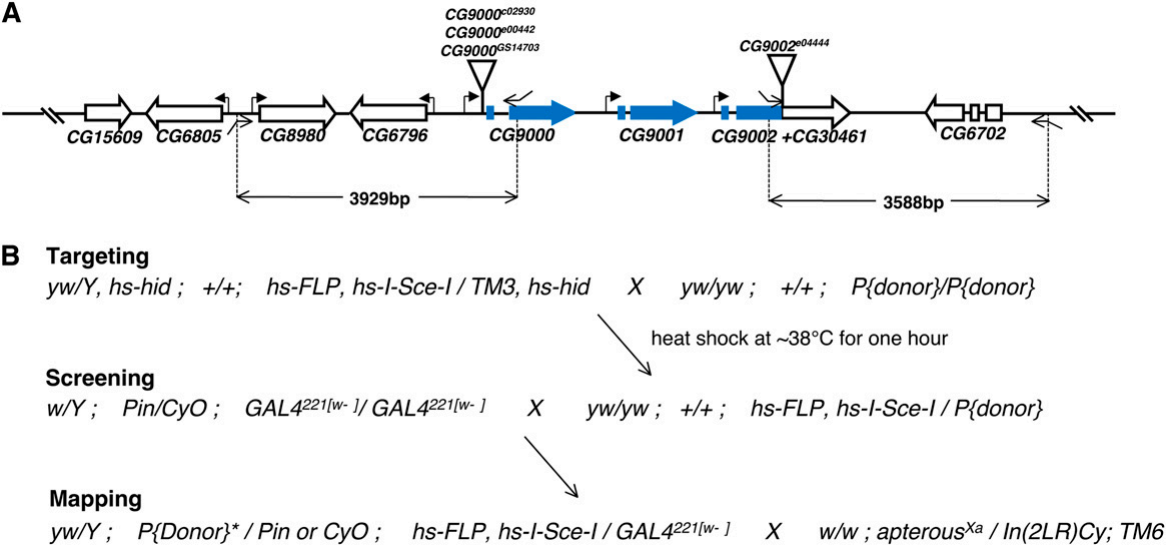
*CG9000* genomic region and crossing scheme for targeted deletion of the tandem *Drosophila STE24* paralogs. (A) Diagram depicting genomic organization of the *CG9000* region, including upstream and downstream genes (not to scale). The three *CG9000* paralogs (*CG9000*, *CG9001*, and *CG9002*) are shaded blue. The arrows with diagonal bends indicate the location of primers that were used in the design of the donor construct for gene deletion. The distances in base pairs below each primer position indicate the length of each flank that was cloned into the donor construct. Right-angled arrows indicate promoter regions for each gene. Inverted triangles indicate the location of P element or PiggyBac insertions. Note that *CG30461* is indicated as dicistronic with *CG9002*, based on evidence from an fused cDNA from the Drosophila Genome Project (DGC:AT28654). (B) Crossing scheme showing the three generations required to isolate a targeted deletion of the *CG9000* region. FLP:Flippase (a yeast recombinase); *I-Sce-1*: a restriction endonuclease; *Pin* is a dominant marker on the second chromosome; *CyO*, *In(2LR)Cy* and *TM3* are balancer chromosomes. *P{Donor}* refers to the donor construct pRK2 into which the regions flanking the *CG9000* cluster were cloned (see *Materials and Methods*). Note that this construct is marked with an asterisk (*) in the third generation, indicating movement from the third chromosome to the second. The use of balancer and Y chromosomes with the apoptosis promoter *HID* driven by heat shock maximizes selection of the correct intermediate genotypes. In addition, the donor construct includes another apoptosis promoter *REAPER* driven by GAL4 in the mapping generation that minimizes selection of false positives (Huang *et al.* 2008).

Primers used to amplify the flanking regions are described in supporting information, Table S1, and their locations in context are indicated in Figure 1A. The flanking regions were amplified from a wild-type stock (*y[1] w[67c23]*) using a high-fidelity Taq from NEB (Phusion M05030S) to minimize the chances of incorporating errors. The flanking regions were cloned into the vector pRK2, which uses a GMR-enhanced *w+* (wild-type red eye) reporter that results in very strong *w+* expression, facilitating selection of successful targeted events. Multiple sequencing runs (University of Arizona Genetics Core DNA sequencing) covering the entirety of both flanks was carried out to ensure fidelity. Targeting constructs were injected by Best Gene (BestGene Inc.), and the three-generation gene deletion procedure was carried out as described in Huang *et al.* (2008) and Figure 1B, except that for the third (mapping) generation, the *w*; *apterous^Xa^/In(2LR)Cy*; *TM6* stock was used.

#### Confirmation of targeted gene deletion:

All primers were ordered from Integrated DNA Technologies. Primers were designed to amplify portions of *CG9000*, *CG9001*, and *CG9002* (Table S1). As a positive control, we used primers for genes in the immediate vicinity: *CG15609* and *CG6805*. Genomic DNA was extracted using 5 Prime ArchivPur DNA Cell/Tissue kit (Cat. No. 2300810). Genomic DNA was tested for integrity and diluted for multiplex PCR with experimental and control primer pairs using NEB Taq (M0267L) with the following amplification conditions for both experimental and control runs: 95°/2 min; followed by 30 cycles of 95°/30 sec, 60°/30 sec, 70°/45 sec; and a final extension 70°/5 min. Products were analyzed on a 2% agarose gel using an Alpha Imager Mini gel documentation unit (AlphaInnotech). This diagnostic procedure was carried out every time a stock was built carrying the triple knockout, and it must be continually performed every few generations in the maintained stocks to ensure no infiltration of recombinant chromosomes that arise from balancer breakdown (see text).

#### Reverse transcriptase PCR:

Total RNA was extracted from relevant genotypes using the Qiagen RNeasy Plus kit (Cat. No. 74134) and up to five micrograms was used in a first-strand synthesis reaction (Superscript III First Strand Synthesis system, Cat. No. 18080051, Invitrogen). Primers used to distinguish between fusion and unique transcripts are described in Table S1. Amplification conditions were as follows for both experimental and control runs: 95°/2 min; followed by 30 cycles of 95°/30 sec, 60°/30 sec, 70°/35 sec; and a final extension 70°/5 min. Products were serially diluted and analyzed on a 2% agarose gel.

#### Rescue constructs:

Gold standard cDNAs for *CG9000*, *CG9001*, and *CG9002* were obtained from the Drosophila Genomics Resource Center (https://dgrc.cgb.indiana.edu/). LD04933 (*CG9000*) was obtained from an embryonic library, whereas AT22982 (*CG9001*) and AT28654 (*CG9002*) came from an adult testis library (http://www.fruitfly.org/EST/faq.html#cdna-1). The cDNAs were amplified with a high-fidelity Taq (Phusion M05030S) and cloned into the Eco*RI* sites of pGEM (Promega PR-A1360). From pGEM, the cDNAs were subcloned into pCaSpeR-hs-ACT (Thummel and Pirrotta 1992) downstream of a heat shock–inducible promoter. Finished constructs were sequenced several times to ensure fidelity and sent to BestGene for injection.

### Molecular evolutionary analysis

#### Sequences and sequence alignment:

We obtained complete coding sequences for the *Drosophila melanogaster* loci *CG9000*, *CG9001*, *CG9002*, *CG7573*, and *CG30461* from FlyBase (Tweedie *et al.* 2009). We further used FlyBase to obtain full coding sequences for the identified orthologs of these genes from the other 11 fully sequenced *Drosophila* genomes. Some sequences were exluded: *CG9001* in *D. simulans*, because no orthologous sequences for this gene could be identified; *CG9002* and *CG30461* in both *D. simulans* and *D.sechellia* due to apparent incomplete assembly; *D. persimilis CG7573* due to apparent incomplete assembly. We used all five *D. melanogaster STE24* paralogs to conduct tblastx searches (Altschul *et al.* 1990) of GenBank’s nr protein database, but we were only able to obtain matches for *CG9000* outside the genus *Drosophila*. We obtained full-length coding sequences of *STE24* orthologs from the outgroup taxa *Anopheles gambiae*, *Aedes aegyptii* (both Diptera), *Tribolium castaneum* (Coleoptera), *Mus musculus*, and *Homo sapiens*. We aligned sequences by codons with the ClustalW algorithm as implemented in MEGA 5.0 (Tamura *et al.* 2011) using default settings.

#### Survey of the outgroup species Rhagoletis pomonella genome for related loci:

We used tblastx (Altschul *et al.* 1990) to search the unassembled whole-genome sequence of *Rhagoletis pomonella* (apple maggot fly, Diptera:Tephritidae) for homologous loci to *CG9000*, *CG9001*, *CG9002*, and *CG7573*. The *Rhagoletis pomonella* genome sequence consists of short 180 bp next-generation Illumina sequence reads with 5–10X coverage (S. Berlocher, J. Feder, and H. Robertson, personal communication). *Rhagoletis* genomic sequence reads that were identified as similar to one of the *D. melanogaster* type I prenyl protease loci in the four separate tblastx searches were separately assembled using Sequencher (Gene Codes Corp.) We then annotated the resulting contigs via blastx searches (Altschul *et al.* 1990) of *D. melanogaster* nonredundant protein sequences in GenBank. We used *CG4852*, the type II *Drosophila* prenyl protease, as a positive control for the possibility that the absence of matching loci in the *Rhagoletis* genome is caused by uneven genome coverage. A search of *Rhagoletis* cDNA contigs (S. Berlocher, J. Feder, and H. Robertson, personal communication; Schwarz *et al.* 2009) produced a close match to *CG9000*, and we combined the *Rhagoletis* cDNA with the short reads to assemble the full CDS of a gene that most likely represents the *Rhagoletis STE24* ortholog. (Contig sequences are available upon request from the authors.)

#### Phylogenetic analysis:

We constructed a phylogeny that explores the relationships of *CG9000*, *CG9001*, *CG9002*, and *CG7573*. In a second phylogeny, we added the highly divergent *CG30461* locus. Both phylogenies were constructed for nucleotide sequences with the “maximum likelihood” method as implemented in MEGA 5.0 (Tamura *et al.* 2011). Additional analyses explored the placement of the *Rhagoletis STE24* ortholog. We used the Tamura-Nei model (Tamura and Nei 1993), included sites with gaps, and used “nearest neighbor interchange” as a heuristic search method. We tested the phylogenies with 500 bootstrap replicates.

#### Genetic distances and relative rates:

We tested differences in evolutionary rates in *D. melanogaster* by Tajima’s relative rate test (Tajima 1993) as implemented in MEGA 5.0. *CG9000* was also tested against *Anopheles STE24* and the *Rhagoletis pomonella* ortholog of *STE24*. *Homo sapiens Zmpste24* served as the outgroup in all tests. We corrected for multiple tests using the Benjamini-Hochberg correction (Benjamini and Hochberg 1995).

#### Detection of negative selection:

We tested the codons of all five *Drosophila* loci for the signature of negative selection using the SLAC method (Kosakovsky Pond and Frost 2005) as implemented in the HyPhy package (Delport *et al.* 2010). To compare the proportion of negatively selected codons, we considered only regions in which the strongly divergent *CG30461* aligned with the other four loci. To optimize the number of aligned codon positions, we excluded *D. simulans* from analysis at all loci due to reasons described above: the *CG9000* region is either highly diverged or incompletely annotated in this species.

### Immunohistochemistry and microscopy

For bright field microscopy, adult males were dissected in 0.7% saline or phosphate buffered saline (PBS) and immediately mounted (unfixed) under 1 mm glass coverslips and examined using phase optics on an Olympus CX41RF compound microscope. Images were collected using an Infinity 2-1C CCD camera. For immunofluorescence microscopy, adult males (for testes) or synchronized L3 larvae (salivary glands) were dissected in PBS, and whole testes or salivary glands were fixed for 20 min in 4% paraformaldehyde (testes) or 15 min in 2% paraformaldehyde (salivary glands), washed several times in PBS-Triton-X (130 mM NaCl; 7 mM Na_2_HPO_4_; 3 mM NaH_2_PO_4_; 10 mM EGTA; 0.1% Triton-X), and blocked in PBS-Triton-X and 3% BSA. Incubation in primary antibody (in PBS-Triton-X + 3% BSA) was overnight at 4°, and secondary for 2 hr at room temperature. Lamin Dm_0_ was detected using ADL84.12 obtained from the University of Iowa Hybridoma center (http://dshb.biology.uiowa.edu/), diluted 1:400. Secondary was goat-anti-mouse Alexafluor 546 from Invitrogen (A21123), used at a dilution of 1:1000. DNA was detected using 10 mM DAPI diluted 1:1000 and added to the first wash after incubation with secondary. Actin was detected using phalloidin-TRITC or phalloidin-FITC at 1:200 (Sigma Cat. Nos. P1951 and P5282, respectively) for 2 hr at room temperature. Fluorescence microscopy was performed using a Zeiss Axioscope A1 compound microscope equipped with fluorescence objectives, LED excitation modules, and filter cubes for DAPI, GFP, and TRITC. Data were collected using a SPOT-cooled CCD camera (Model RT3), and image (channel) processing was carried out in Adobe Photoshop 7. Serial stacking for high-power GFP-only images was carried out using Combine ZP (Open Source Image Stacking Software by Alan Hadley).

## Results

### Deleting *CG9000*, *CG9001*, and *CG9002* from the genome using ends-out gene targeting

Gene duplications pose special problems for biological analysis due to the possibility of redundant function. In *Drosophila*, duplication of the *STE24* function appears to have taken place multiple times, leading to the present set of three tandem paralogs (*CG9000*, *CG9001*, and *CG9002*) on the second chromosome that possess the canonical HEXXH zinc ion binding active site, and an additional potential paralog on the third chromosome, *CG7573*, which demonstrates sequence changes at this active site (Figure S1; Corrigan *et al.* 2005). There are a number of viable and fertile transposable element insertions stocks in all the *Drosophila STE24* paralogs (except *CG9001*), and the smallest deficiency for the region (*Df(2R)Exel6065*) (Tweedie *et al.* 2009) takes out 37 additional genes (including several known essential genes). Therefore, to accurately determine the loss-of-function phenotype for the most conserved *STE24* functions in *Drosophila*, it was necessary to remove all three genes by a targeted process. Ends-out gene targeting (Rong and Golic 2000; Gong and Golic 2003) is a proven methodology; however, the advantage of targeting is labor intensive, because to obtain a successful targeted deletion event, many tens of thousands to hundreds of thousands of flies must be screened (Venken and Bellen 2005). As a result, a number of innovations have been developed, and we elected to use a recently refined protocol that includes two rounds of negative selection, minimizing labor by facilitating the identification of true positive events (Huang *et al.* 2008). The details as applied to the current experiment are outlined in *Materials and Methods* and Figure 1, which includes a diagram of the genomic region containing the three tandem prenyl protease genes (Figure 1A). These genes span a genomic region of approximately 6.2 kb, and *CG9002* appears to be fused to another gene transcribed in the same direction (*CG30461*). To avoid disrupting any putative regulatory sequences for genes in the vicinity, we elected to delete just downstream of the start of transcription for *CG9000* and just prior to the termination site in *CG9002*. We left *CG30461* behind due to the fact that it does not appear to encode a prenyl protease function. We later learned that *CG30461* is very likely a highly diverged (and truncated) additional duplication of from the *CG9000* cluster, a probable pseudogene. Figure 1B depicts the crossing scheme used to generate the targeted deletion.

The results are depicted in Table 1. Of the potential true positives (indicated by the wild-type red eye color (*w+*) reporter), females outnumbered males by approximately three to one. To establish breeding stocks, males were selected for further analysis, and approximately half were false positives. The remaining stocks were tested by PCR for the loss of the three *STE24* homologs (see *Materials and Methods* and Figure S2 for details) resulting in a final targeting rate of eight confirmed independent events out of approximately 125,000 flies scored or about 1 in 5,000 gametes (corrected to include putative female events that were not characterized). Of those eight, three were singled out for further experimental analysis: 5R8, 6AR2 and 7AR7.

**Table 1:** Results from ends-out targeted gene deletion screen for *CG9000*, *CG9001* and *CG9002*

Total No. of F2 Flies Scored	Total No. of F2 *w^+^* Males	Total No. F2 *w^+^* Female	Total No. of *w^+^* Males Segregating with Chromosome II	Total No. of Confirmed Triple Gene Knockouts
125,000*^a^*	110	353	40	8*^b^*

a Estimate based on 26 F2 (screening generation) trays, approximately 4800 flies scored per tray.

b Because entire trays were scored en masse, six lines had to be independent events (isolated from separate trays), and two were probable but not proven independent events (isolated from the same tray).

Almost as soon as the triple knockout deletion chromosomes were identified, we began to lose them. The triple knockout deletion was initially maintained in a heterozygous state, paired with a balancer for the second chromosome. Balancers are multiply rearranged chromosomes designed to suppress meiotic recombination. However, sometimes balancers fail, a process known as balancer breakdown. The balancer we used to isolate the triple knockout was in fact a “weak” balancer called *In(2LR)Cy* (Tweedie *et al.* 2009) consisting of a single inversion in each arm of the metacentric second chromosome. These two rearrangements were evidently not sufficient to stabilize the *CG9000* region, and after a single generation, the *w+* (red) eye color reporter that marked the deletion began to disappear from all eight triple knockout stocks. The loss of the reporter correlated with the reappearance of the three *CG9000* genes as determined by PCR (data not shown), presumably due to recombination in the maternal germline between the triple knockout chromosome and the balancer (there is no meiotic recombination in *Drosophila* males). This problem was temporarily rectified by recovering the *w+* heterozygous male siblings and crossing them to stronger balancers (with more rearrangements). On two occasions, we recovered a balancer onto which the triple knockout had been recombined (the reciprocal product), which was verified by PCR, proving that the source of breakdown is in fact from the balancer (see Figure S3). In sum, we have successfully deleted the three tandem type I prenyl protease genes from the *Drosophila* genome and have determined that they reside in a region that is particularly susceptible to balancer breakdown.

### Loss of *Drosophila STE24* function causes a male fertility defect

**Figure 2:**
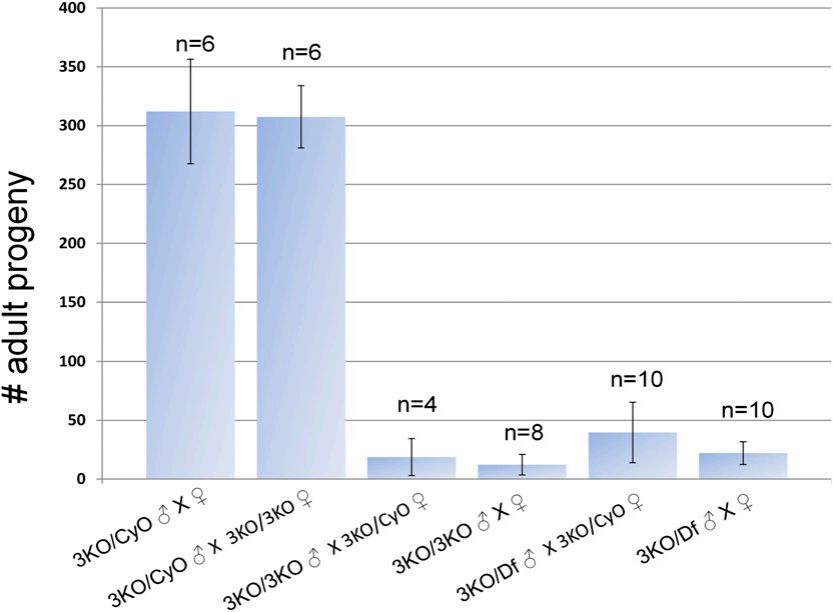
Fertility test data for the triple knockout genotypes removing the three tandem *STE24* paralogs. A fertility defect reveals itself only when males are homozygous for the triple knockout. “n” refers to the number of times a particular cross was set up. For each cross, five virgin males and females were mated to each other, and the crosses were turned into fresh vials every three to five days. Scoring of adult progeny was stopped after three vials. In all cases, where homozygous triple knockout male parents were involved, no progeny were produced past the second vial and usually not past the first vial. Df refers to *Df(2R)Exel6065*, which removes the three *CG9000* genes and 37 additional genes.

Heterozygous triple knockout males and females were perfectly fertile and viable. However, it was not possible to construct a homozygous population, and this was true for all eight confirmed events. The most likely explanation was a fertility defect. To determine which sex was affected, we designed a genetic experiment consisting of four defined crosses in which heterozygote and homozygote males and females were crossed in all possible combinations. The results are depicted in Figure 2. All four crosses were carried out several times for each of three independent triple knockout genotypes (5R8, 6AR2, and 7AR7), which all behaved similarly, so the data were pooled. Figure 2 shows that the fertility defect is confined to the males: homozygous females are indistinguishable from heterozygous females in terms of fertility. Five triple knockout males can typically produce about 17 progeny; under the same conditions, their heterozygous siblings produce at least 250. As an additional test for genetic background, males that were transheterozygous for a deficiency that removes all three genes (*Df(2R)Exel6065*) and the triple knockout chromosome were also tested: they produced on average 22 progeny, a slight but statistically insignificant increase.

Finally, to determine the penetrance and expressivity of the phenotype, 15 individual triple knockout males were each crossed to 4 homozygous female siblings and scored for adult progeny (see Table S2). About a third of this pool of males was completely sterile, and of the remainder, the average number of offspring was 5 (mode = 2; largest number of progeny from a single male = 16). Therefore, the male fertility defect is completely penetrant, but it shows variable expressivity.

### *Drosophila STE24* triple knockout has a modest effect on male viability, and the fertility defect is age dependent

As *Zmpste24* has been implicated in a disease of premature aging in humans, we wanted to know whether loss of the three tandem paralogs in *Drosophila* affected life span. Although female life span appeared unaffected by the triple knockout, we did note a statistically significant reduction (approximately 20%) in triple knockout male life span relative to heterozygous siblings (Figure S4).

**Figure 3:**
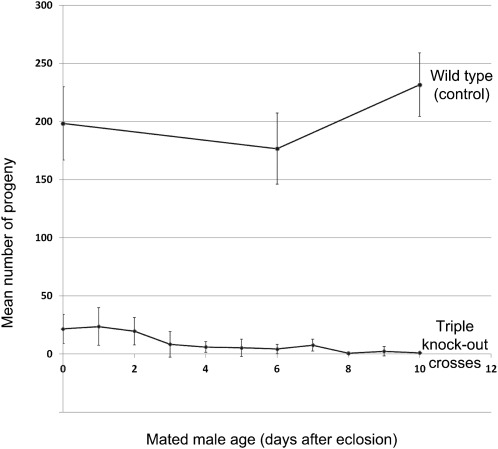
The fertility defect in triple knockout males is age dependent. Each data point represents the number of progeny resulting from a cross between five virgin homozygous males and females. The virgin male ages were as indicated (after eclosion) before exposure to the females. Fertility drops off to zero after about a week. Wild-type controls were set up for only three data points.

To determine whether the sterility defect was constant throughout the life of the male or whether it was a function of age, we performed a fertility curve analysis, in which five newly eclosed virgin homozygous males were mated to five (1–2 days old) virgin homozygous females in a series of crosses in which the virgin males differed in age by one day. The crosses were performed in triplicate, with each triplicate an independent but otherwise identical triple knockout line (5R8, 6AR2, and 7AR7). There were no differences between the genotypes, so the data were pooled (Figure 3). Triple knockout male fertility peaks at around day 1, but by day 10, the males are effectively sterile. Therefore, the loss of the three *STE24* paralogs causes an age-dependent fertility defect in males only.

### Phylogenetic analysis of the *Drosophila STE24* genes reveals additional paralogs in the genome and sequence diversification

The *Drosophila STE24* function has a demonstrated role in the male germline, and it appears to have undergone serial duplications in the genome. These two observations strongly suggest an evolutionary process that has led to a division of labor among the paralogs with subfunctionalization in the male germline. There is substantial literature describing duplicated genes with sex-specific evolutionary consequences (Torgerson and Singh 2004; Hollaway and Begun 2004; Haerty *et al.* 2007; Ding *et al.* 2010). It was therefore of interest to perform a phylogenetic analysis to examine the evolutionary history of the type I prenyl protease genes in *Drosophila* to test the hypothesis that they originated via duplication, and show evolutionary patterns that are indicative of having acquired a new function in the germline. Our analysis was facilitated by the availability of the recently sequenced and annotated additional 11 *Drosophila* species (Drosophila 12 Genomes Consortium, 2007), which add power to evolutionary inferences.

**Figure 4:**
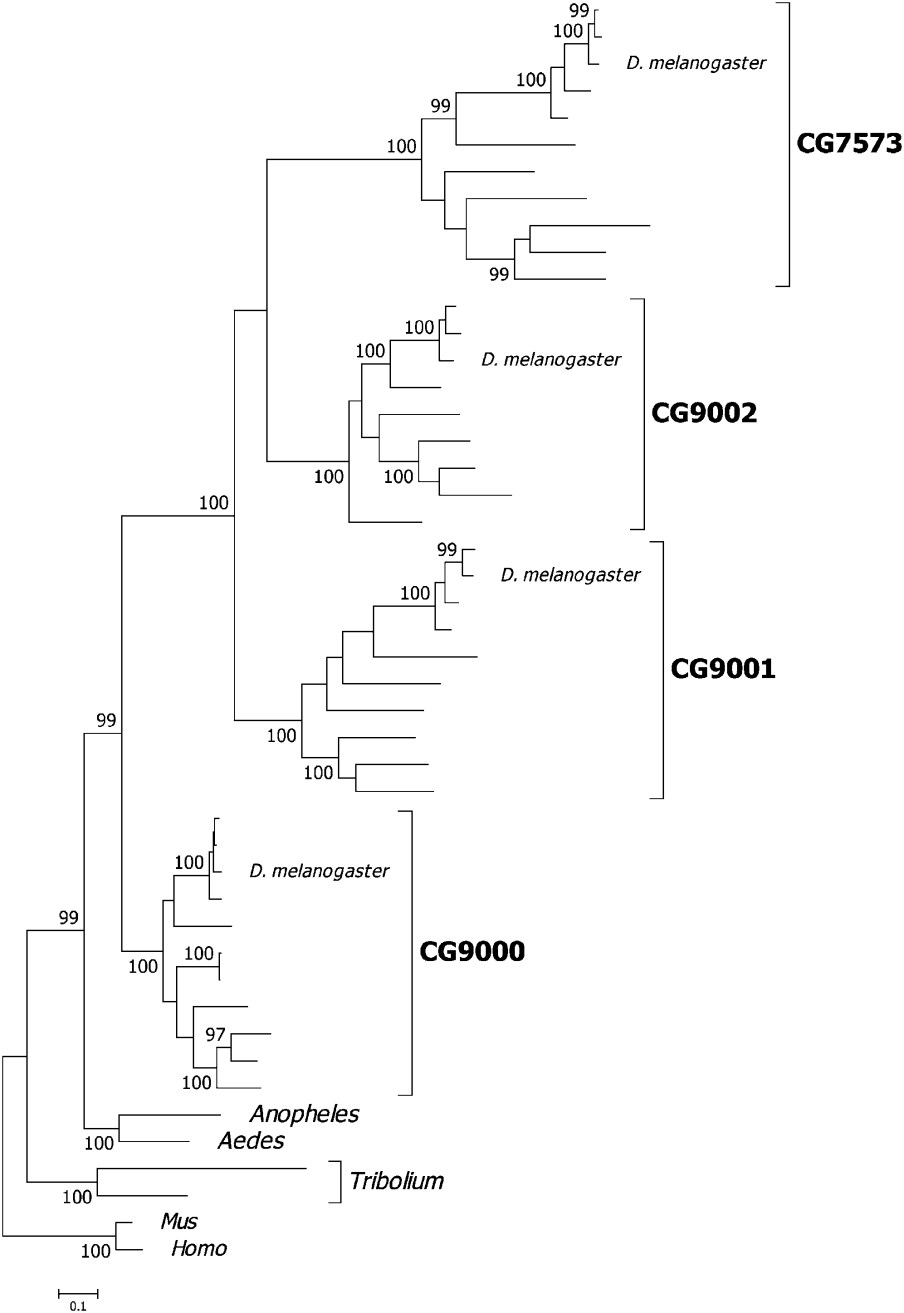
Maximum likelihood phylogeny for *CG9000* and paralogs. CG numbers for each gene denote the clades of *Drosophila* species for the respective locus. The figures above the nodes are the results from bootstrap analysis with 500 replicates (only bootstrap values ≥ 95 are shown). The scale is given as substitutions per site. (See Figure S6 for a similar analysis that includes *CG30461*.)

A phylogenetic analysis revealed that all *Drosophila* loci form a well-supported monophyletic clade with a bootstrap (confidence) value of 99% (Figure 4). The *Drosophila* clade is further subdivided into equally well supported clades for *CG9000*, *CG9001*, *CG9002*, and *CG7573*. This pattern is consistent with a diversification of the *STE24* prenyl protease gene in a common ancestor of the 12 *Drosophila* species. The phylogenetic order suggests that *CG9001* is ancestral to *CG9002* and *CG7573* (this order is supported in 89% of test replicates). This pattern implies a sequential duplication of *CG9000* giving rise to *CG9001*, which in turn gave rise to the ancestor of *CG9002* and *CG7573*. Duplication of the *STE24* ortholog can only be found in one other species, also an insect, the flour beetle *Tribolium*, which based on the phylogenetic analysis, represents a separate, independent event.

To better resolve the time frame during which these gene duplications might have occurred, we searched the as-yet unassembled (5–10X) coverage genomic sequence reads of the entire *Rhagoletis pomonella* (apple maggot) genome **(**S. Berlocher, J. Feder, and H. Robertson, personal communication**)**. *Rhagoletis* (family Tephritidae) and *Drosophila* both belong to the schizophoran clade of flies that shared a common ancestor as recently as 65 million years ago (Grimaldi and Engel 2005). We were able to obtain a virtually complete *Rhagoletis* *CG9000/STE24* coding sequence; however, when we queried the *Rhagoletis* genomic database with *Drosophila* *CG9001* and *CG9002* coding sequences, we obtained matches that all corresponded to the *Rhagoletis* *CG9000/STE24* ortholog **(**Table S3). In addition, a query of the *Rhagoletis* genomic database using *Drosophila CG7573* recovered no sequences, whereas the same procedure using the *Drosophila* coding sequence for the type II prenyl protease *CG4852* easily produced a *Rhagoletis* ortholog, indicating sequence coverage from the *Rhagoletis* database was sufficient. Therefore, *Rhagoletis*, like almost all other species, appears to possess single genes for the type I and type II prenyl proteases.

Because *CG30461* shows no significant homology to any of the *STE24* paralogs, and it has been classified as an unrelated function. However, when run in a carefully “manually” curated ClustalW alignment with *CG9002* *Drosophila* orthologs, *CG30461* shows sufficient homology to be a potential further duplication. When *D. melanogaster CG9002* and *CG30461* amino acid sequences (excluding indels) are aligned (a total of 190 amino acids), these two sequences demonstrate 16% sequence identity and 32% sequence similarity calculated from the ClustalW alignment in Figure S5. *CG30461* appears to terminate immediately prior to the presumed active site of the enzyme (HEXXH, Figure S5). This truncation and the high degree of sequence divergence may account for the failure to previously annotate *CG30461* as a related prenyl protease gene. When we included the highly divergent *CG30461* in the phylogenetic analysis, it formed a separate, well-supported clade that is part of the clade containing the paralogs of *CG9000* (Figure S6), showing that it, too, likely originated via duplication in a common ancestor. In addition, we have confirmed by RTPCR that *CG30461* can be expressed both as a fusion transcript with *CG9002* and as an independent transcript in a triple knockout genetic background (Figure S7).

The branch lengths in both phylogenies further show that *CG9001*, *CG9002*, and *CG7573* have evolved at approximately twice the rate of *CG9000*. This difference in evolutionary rate is statistically significant when tested rigorously solely in *D. melanogaster*. In contrast, the *D. melanogaster CG9000* gene is not evolving any faster than the two other dipteran species for which sequence information is available (*Anopheles gambiae* and *Rhagoletis pomonella*, Table S4). Therefore, the increase in evolutionary rates after duplication is consistent with a model in which the paralogs became refunctionalized for a germline-specific role. Evolutionary rates were even higher in *CG30461* (Table S4), a finding that is consistent with a much lower proportion of negatively selected codons under purifying selection (approximately 8%) when compared with the other loci (17–42%, Table 2).

**Table 2:** Proportion of codons under negative selection

Locus	Negatively Selected Codons	Total No. of Codons	Proportion
*CG9000*	68	163	0.42 (a)
*CG9001*	34	163	0.21 (b)
*CG9002*	54	163	0.33 (a, b)
*CG7573*	27	163	0.17 (b, c)
*CG30461*	13	163	0.08 (c)

Analysis with the SLAC codon-based maximum likelihood method. Letters a–c indicate statistically significantly different proportions (Fisher exact test with Benjamini-Hochberg correction of alpha = 0.05 for multiple tests).

### Independent germline rescue with *CG9000*, *CG9001*, or *CG9002* supports the evolutionary analysis of diverging functions

One way to determine the contribution to fertility of the three tandem prenyl protease genes is to restore them to the triple knockout genotype by germline transformation, and then test for rescue of fertility. A variety of rescue strategies are available, including genomic rescue with a single construct or multiple transgenes under heat shock or GAL4 induction (Brand and Perrimon 1993). Given the recombinatorial instability of the region that would render rescue stock construction challenging, we elected the simplest method that enabled independent assessment of each prenyl protease gene separately under heat shock induction (pCaSpeR-hs-act) (Thummel and Pirrotta 1992). Constructing these rescue stocks meant introducing another balancer into the genotype to ensure the transgene would be present, which enhanced the balancer breakdown issue we had noted earlier. Under these circumstances, there were no balancers strong enough to overcome this breakdown, which has been described as an interchromosomal effect (Schultz and Redfield 1951). This means that a test male (bearing a rescue transgene) that appears homozygous for the triple knockout chromosome might in fact be heterozygous, having received a recombinant gamete from his (balanced) mother. Therefore, we carried out the rescue experiments using single males that were heat shocked from 6 to 8 days after egg lay, and then crossed to their homozygous virgin siblings (bearing no rescue transgenes). The presence or absence of the prenyl protease genes were assessed in all progeny and parents of every cross by PCR (Figure S8). We chose the 6–8 day time frame because earlier heat-shock regimens appeared to negatively affect cross viability. Because the heat-shock promoter used in the germline transformation vector pCaSpeR-hs-act is reported to work only in early (mitotic) gonial cells (Michaud *et al.* 1997), we were relying on perdurance of the protein to supply the required dose of prenyl protease for rescue.

The data are shown in Table 3. Two classes of rescue were obtained. In the first class (Class I in Table 3), each male was shown by PCR to be a true homozygous triple knockout with a rescue transgene present (Figure S8). In none of these males was fertility fully restored; however, it is possible to reject the null hypothesis (that the rescue transgene had no effect) for crosses in which *CG9001* or *CG9002* expression was restored by heat shock. In these crosses, exceptional males (males that could produce 10 or more progeny) appeared at a higher frequency in a smaller sample size relative to controls. In addition, induction of either of two different *CG9001* transgenes resulted in triple knockout males that were now capable of producing between 30 and 40 progeny (two males out of 18 and 15, respectively).

**Table 3:** Partial and complete rescue of the triple knockout fertility defect

Male Genotype	N*^b^*	True 3KO Males*^c^* (Class I)	Males with 0–9 Progeny	Males with >10 Progeny	*P* Value*^d^*	*P* Value*^e^*	Recombinant Males*^f^* (Class II)	Recombinant Males with Wild-Type Fertility*^g^*
Control: 3K0*^a^*	72	72	67	5	NA	NA	NA	NA
3KO-9000-5M	28	19	16	3	0.226	0.450	9/28 = 32%	9/9
3KO -9000-10M	16	10	9	1	0.728	0.764	6/16 = 38%	6/6
3KO-9001-1M	27	18	11	7	>0.001	0.001	9/27 = 33%	9/9
3KO-9001-2M	21	15	8	7	>0.001	>0.001	6/21 = 29%	6/6
3KO-9002-2M	19	17	12	5	0.00833	0.027	2/19 = 11%	2/2
3KO-9002-5F	11	7	5	2	0.05458	0.220	2/11 = 18%	2/2

a Triple knockout homozygote (3KO) with or without rescue transgene.

b Total number of individual males tested.

c Verified by PCR (Figure S8). If true 3KO/3KO, only rescue transgene present. If recombinant, all three genes back from balancer.

d Chi-square test.

e Chi-square test with Yates correction for sample size.

f Males that received a recombinant gamete from mother in which three paralogs returned from the balancer.

g Wild-type fertility arbitrarily set at greater than or equal to 100 progeny from a single male.

In the second rescue class (Class II in Table 3), the males were heterozygous as determined by PCR, having received a recombinant gamete from the maternal parent that restored the three paralogs (Figure S8). These males were fully fertile. On the basis of these results, we determined that females heterozygous for the triple knockout and the balancer produced recombinant gametes at a rate of 27% on average. Although we cannot pinpoint the precise location of the crossover events, our rescue data show complete restoration of fertility as a result of 34 independent recombinant gametes, all of which had in common the restoration of all three genes. Therefore complete rescue of the fertility defect arising from a lack of the type I prenyl protease in the male germline occurred only in the presence of all three genes in their native genomic configuration.

There are no antibodies that can be used to determine whether and to what level the transgenes are expressing. However, the evidence for partial rescue strongly argues the transgenes are expressing, which is supported by the interesting finding that the transgene stocks alone (17 lines tested, multiples for each paralog), even at room temperature, are predominantly homozygous lethal or semilethal (data not shown). Therefore, we conclude that *CG9001* and *CG9002* are better able independently to rescue a knockout for all three tandem prenyl protease functions in *Drosophila* and that *CG9000* may require higher levels of expression, or else, it encodes an enzyme that no longer functions efficiently in the germline.

### Loss of the three tandem *STE24* functions in *Drosophila* causes a post-meiotic spermatogenesis defect

**Figure 5:**
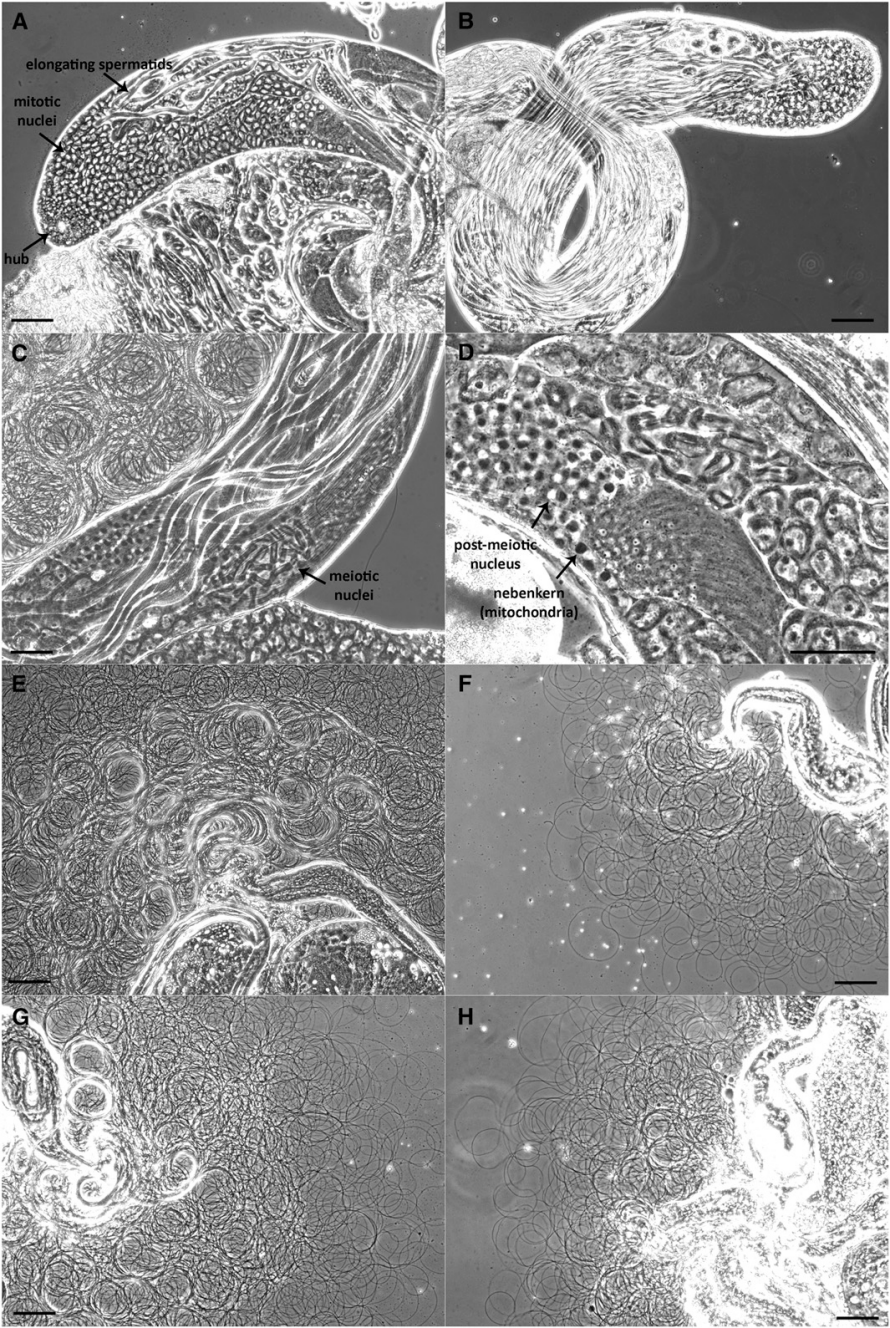
No changes in the principal stages of spermatogenesis in triple knockout *vs.* control genotypes. Phase contrast images of testes dissected from age- and condition-matched heterozygous or wild-type (A, C, E, G) and homozygous triple knockout (B, D, F, H) males. (A, B) Apical sections of testes from 10- to 15-day-old males, showing gonial and mitotic spermatogonia, elongated spermatids, and “waste bags,” spherical structures containing excess cytoplasm squeezed out of the spermatids during elongation. (C, D) Midsections of testes from 21-day-old males, showing meiotic spermatids, postmeitoic (round, phase light) spermatids, elongating spermatids, and onion stage mitochondria (nebenkern, phase dark). (E, F) Dissections releasing mature sperm from 3-day-old males. Note lesser amounts of sperm from triple knockout males. (G, H) Dissections releasing mature sperm from 8- to 10-day-old males. Note that although there is still less sperm in the triple knockout homozygote (H) than in the wild-type (G), at this point the triple knockouts are virtually completely sterile. Scale bars: 100 µM (A, B, E, F, G, H) and 50 µM (C, D).

Triple knockout males are almost completely sterile, and there were no obvious defects in courtship and mating (Greenspan and Ferveur 2000). Because homozygous triple knockout males are fertile for only a narrow window early in their adult life, they can make functional sperm. But do they simply make less of it, or do they make larger quantities of defective sperm? To establish when during spermatogenesis type I prenyl protease function might be required, we carried out phase contrast microscopy to examine all the stages of spermatogenesis in homozygous triple knockout males and their heterozygous siblings or wild-type controls. Each testis is a long coiled tubular organ, with a stem cell niche at the apical tip. Stem cells divide asymmetrically to produce a gonial cell that undergoes four incomplete mitotic divisions, resulting in a group of 16 interconnected primary spermatocytes encased in a cyst composed of two somatic cells. The cysts of maturing spermatocytes gradually move down the length of the testes as the spermatocytes undergo meiosis, followed by dramatic remodeling events that lengthen the spermatocyte nuclei, coalesce the mitochondria, and build flagella. Ultimately, 64 meiotic products mature into full-length individualized spermatids (Fuller 1993; Zhao *et al.* 2010). As can be seen in Figure 5, there are no notable differences in any of the principle stages when homozygous triple knockout males are compared with their heterozygous siblings (or wild-type). In addition, there appeared to be no reduction in premeiotic cell types as the males aged, arguing against a stem-cell defect. We did notice that sperm spilled (by dissection) from the triple knockout testes was less abundant than sibling controls (Figure 5, E and F). Interestingly, the sperm abundance seemed unchanged, regardless of male age. Compare Figure 5, E and F, in which sperm was spilled by dissection in 3-day-old males, with Figure 5, G and H, where the same procedure was carried out on 8- to 10-day-old males. At this time point, the homozygous triple knockout males are virtually sterile, but their capacity for producing mature sperm appears to be unchanged.

**Figure 6:**
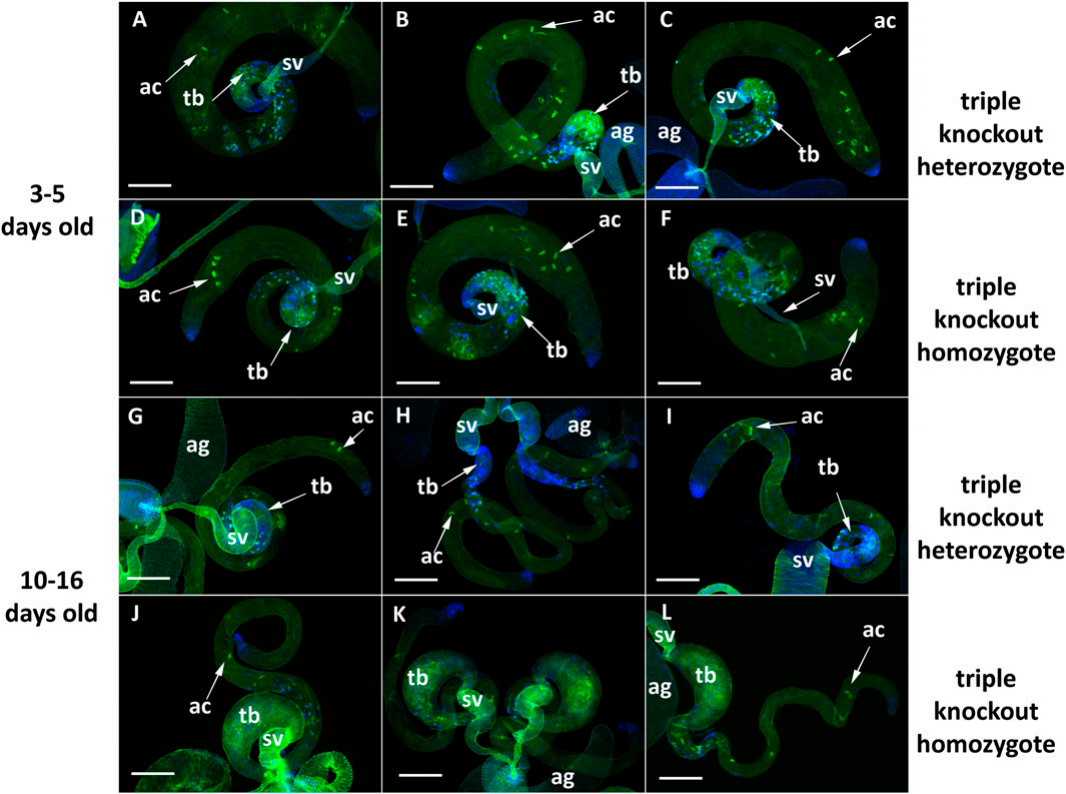
Dissected testes from age- and condition-matched male siblings from fertility crosses (see Figure 2). DAPI stains DNA and phalloidin-FITC was used to highlight actin structures. (A–C) Triple knockout heterozygotes, 3–5 days old. (D–F) Triple knockout homozygotes, 3 to 5 days old. (G–I) Triple knockout heterozygotes 10–16 days old. (J–L) Triple knockout homozygotes 10–16 days old. ac, actin cone (comprising the individualization complex); ag, accessory gland; sv, seminal vesicle; tb, testis base. Scale bar: 200 µM.

To see whether there were defects associated with particular proteins that might explain the fertility defect, we used fluorescence microscopy to determine whether the sterility could be explained by a failure to produce continuous quantities of germ cells or whether loss of a type I prenyl protease function caused a spermatid elongation or individualization problem. For analysis of germ cells, we confirmed our phase contrast data by using a constructed stock that marked the mitotic germ cells with GFP (Sano *et al.* 2002). No reduction in premeiotic germ cells was observed over a three-week period, long past the stage when triple knockout males are no longer fertile (data not shown). For individualization and spermatid elongation, we used phalloidin to mark the clustered group of actin-based cones (the individualization complex) that collect at the base of the spermatid nuclei and act to squeeze excess cytoplasm from the elongating spermatids, ultimately separating the interconnected spermatids into individualized mature sperm (Fabrizio *et al.* 1998). Male fertility defects that fall into this category include a paucity of individualization complexes, aberrant nuclear morphology, and scattering of actin cones (Fabrizio *et al.* 1998; Texada *et al.* 2008; Anderson *et al.* 2009). For fluorescence experiments, we dissected homozygous and heterozygous males from the fertility crosses described in Figure 2, thus ensuring synchronized aging, and identical maintenance and mating conditions. Figure 6 shows a representative selection of heterozygous and homozygous triple knockout testes dissected after 3 to 5 days (A–F), and 10 to 16 days (G–L). We observed two principle phenotypic differences between triple knockout males and their heterozygous siblings, and both phenotypes were age dependent. First, by counting mature individualization complexes (clearly distinct in the upper length of the testis as opposed to the cluster of maturing complexes located at the base of testes near the seminal vesicles), we observed a difference in the number of mature individualization complexes as the flies aged. We saw no difference in age-matched heterozygous or homozygous young males, but older triple knockout males showed a more than 3-fold reduction in mature individualization complexes relative to their age-matched heterozygous siblings (Figure S9). We did not see any scattering of actin cones above a background level also visible in wild-type or heterozygous males regardless of age. Therefore, we conclude that loss of the *Drosophila STE24* tandem paralogs does not affect the formation of the individualization complex *per se*, but it might play a role in either the preceding spermatid elongation or subsequent individualization process. Second, we observed an age-dependent gradual expansion or swelling at the base of the testes where they join the seminal vesicles, in which elongated spermatids appeared improperly coiled (Figure 6, J–L). This swollen phenotype became visible on about day 6, which is the approximate time point when a transition to virtually complete sterility is observed (Figure 3).

**Figure 7:**
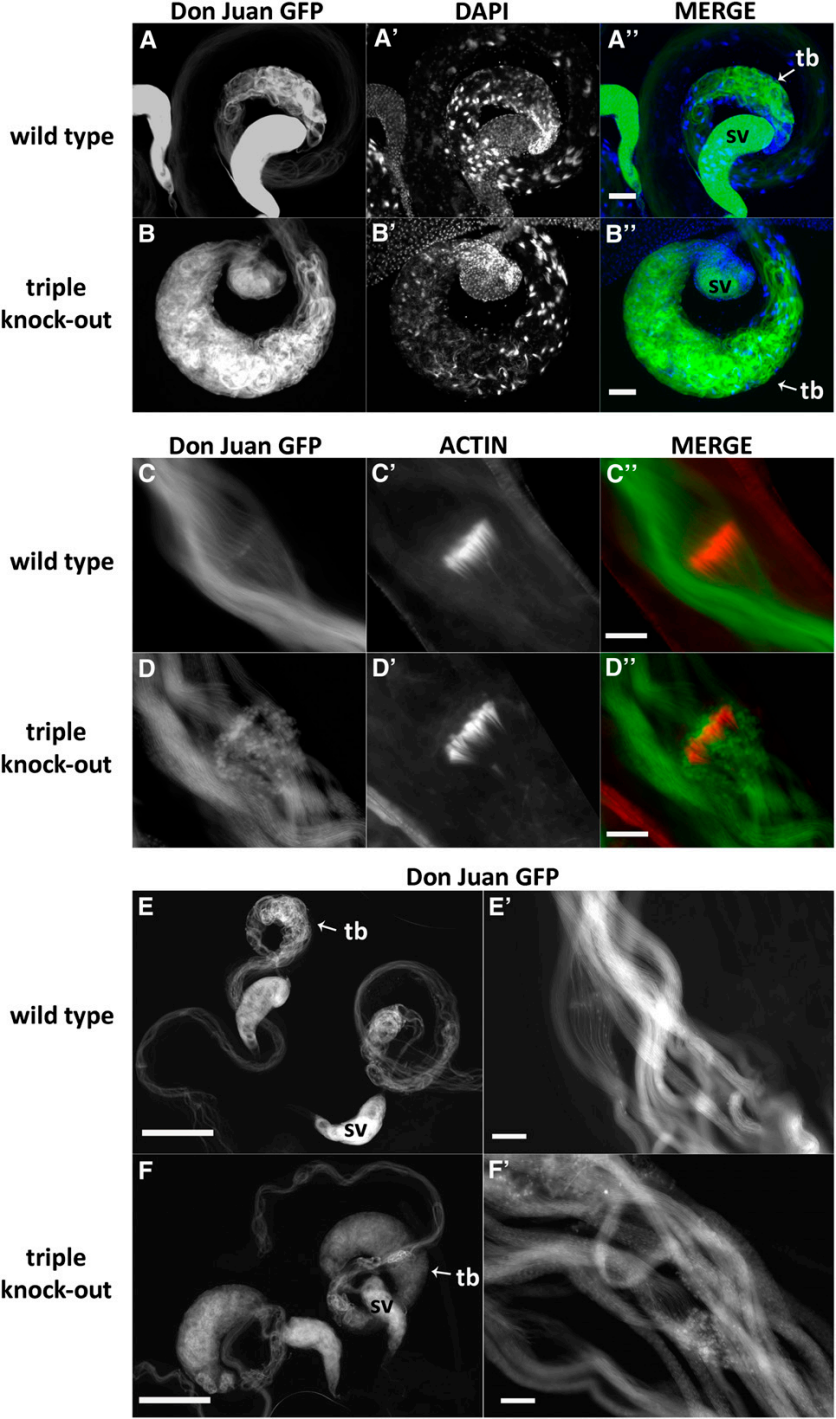
Individualization complexes form normally in triple knockout flies, but elongated spermatids exhibit a cytoplasmic defect. Fluorescence images for heterozygous and homozygous triple knockout spermatids. All males had been mated and were between three and four weeks old. (A, B) Low-power images (20X; scale bar, 50 µM) showing Don Juan–GFP localization in the proximal region of the testes and seminal vesicles. Note that the Don Juan–GFP triple knockout stock was built over a reciprocal translocation (*apterous^Xa^*) between the second and third chromosomes (to minimize balancer breakdown), which means that the homozygous triple knockout males have in fact a double dose of Don Juan–GFP relative to their siblings, yet the heterozygotes consistently fluoresced much more brightly under identical camera settings, supporting the conclusion that far less mature sperm is transported into the seminal vesicles in the triple knockout (see also Figure S10). (C, D) Actin-based individualization complexes (scale bar, 20 µM) are labeled with phalloidin-TRITC, and sperm flagella are labeled with Don Juan–GFP. Although the individualization complex looks normal in both genotypes, the triple knockout exhibits a grainier appearance of GFP. This grainier appearance is more obvious in GFP-only images for the same genotypes: E (scale bar, 200 µM) and E′ (scale bar, 20 µM) are from heterozygous males; F and F′ (same scaling) are from homozygous triple knockout males. sv, seminal vesicle; tb, testes base.

To better visualize the location of the mature spermatids in the testes and storage structures we constructed stocks that expressed a Don Juan–GFP fusion protein in a triple knockout background. Don Juan is a protein that is specifically expressed in elongated spermatids and mature sperm, where it localizes with the sperm tail, possibly with the uniquely structured mitochondrial derivatives that are arrayed along the entire length of the spermatid flagellum (Santel *et al.* 1998). As the triple knockout phenotype was indistinguishable in younger heterozygous and homozygous male siblings, older males were examined for Don Juan fluorescence to determine how loss of the type I prenyl protease might affect spermatid morphology. Figure 7, A and B confirms that the elongated spermatids in triple knockout males accumulate in the base of the testes, and they appear disordered or improperly coiled. Fluorescence for Don Juan is relatively unchanged throughout the length of the testes in both triple knockout homozygotes and heterozygotes, but it is consistently brighter in the seminal vesicles of the triple knockout heterozygotes, indicating less sperm in the seminal vesicles of homozygous triple knockout males (see also Figure S10). Figure 7, C and D show how the individualization complex forms on the sperm tails in both the triple knockout and a heterozygous sibling (or wild-type). The assembly of the individualization complex appears relatively unaffected by loss of the tandem *STE24* paralogs, but there is a distinct difference in the way the Don Juan–GFP protein localizes on the elongated spermatids. While a mixture of grainy and smooth GFP localization can be seen in both genotypes, the grainy appearance predominates in the triple knockout. This graininess is most evident in GFP-only images (Figure 7, E and F).

Finally, as the human Ste24p ortholog Zmpste24 shows strong evidence of processing prenylated lamins (Corrigan *et al.* 2005), we investigated the possibility that the *Drosophila* paralogs might also play a part in processing lamins. Like humans, *Drosophila* expresses both a prenylated lamin (Lamin Dm_0_) and an unprenylated lamin (Lamin C) (Riemer *et al.* 1995; Schulze *et al.* 2005). It is unclear how these lamins relate to A and B type lamins in humans (Erber *et al.* 1999), but specific mutations in the *Lamin Dm_0_* gene show fertility defects in both sexes (Lenz-Bohme *et al.* 1997). Lamin Dm_0_ localization is unaffected in a triple knockout genetic background, as visualized by immunofluorescence *in situ* staining of salivary glands and testes (Figure S11) with anti-Lamin Dm_0_.

## Discussion

*Drosophila* has uniquely diversified its complement of type I prenyl proteases, possessing a tandem array of three paralogs, which are identified by the canonical HEXXH active site, and two additional paralogs, one of which shows substantial sequence divergence at this site (*CG7573*) and another that appears to have acquired a stop codon just prior to the active site (*CG30461*). We have generated the first targeted deletion of the three tandem *Drosophila* type I prenyl proteases, *CG9000*, *CG9001*, and *CG9002*. The targeted triple gene deletion reported here also represents a successful implementation of a refinement of the ends-out gene targeting methodology that has been used routinely for single gene deletions and more rarely for multiple gene deletions (Matsuo *et al.* 2007; Huang *et al.* 2008; Gong and Golic 2004; Venken and Bellen 2005).

Similar to yeast, loss of *Drosophila* type I prenyl protease function causes a fertility defect. Females are totally unaffected by loss of the three tandem *STE24* orthologs, but males show a sharp reduction in fertility, which, while fully penetrant, varies in expressivity. Male germline specificity is also supported by the finding in FlyAtlas in which *CG9000* is shown to be expressed in a wide range of tissues and developmental stages, whereas *CG9001* and *CG9002* are both shown to be upregulated solely in the testis (Chintapalli *et al.* 2007). In addition, *CG9001* and *CG9002*, but not *CG9000*, are underexpressed in meiotic-arrest mutants, supporting their role in spermatogenesis (H. White-Cooper, personal communication). Triple knockout males also show a decrease in viability relative to their heterozygous siblings, but the reason for this is not clear. Connections between fertility and life span have been reported [*e.g.* Hsin and Kenyon (1999)], and further exploration involving the prenylation pathway in the context of life span may be of interest.

In *Drosophila*, diversification of the type I prenyl proteases represents another example of gene duplication with specific functions devoted to the male germline (Ranz *et al.* 2003; Torgerson and Singh 2004; Ding *et al.* 2010). This hypothesis is supported by our data compiled on evolutionary rates, which demonstrate that although *CG9000* remains highly conserved, *CG9001* and *CG9002* (and *CG7573*) are evolving at a faster rate in the 12 sequenced *Drosophila* species. When each gene is added back to the triple knockout genotype one at a time, no rescue is complete, but the data provide evidence of divergent functions between the highly conserved *CG9000* and the more rapidly evolving *CG9001* and *CG9002*. Collectively, these data argue that *CG9000* has undergone serial duplications before the diversification of the *Drosophila* genus, and that *CG9001* and *CG9002* have experienced evolutionary change that has resulted in a specific function in the male germline. The uncertain placement of the *Rhagoletis pomonella STE24* ortholog in the phylogenetic tree makes it difficult to determine whether the origin of the *CG9000* paralogs pre- or postdates the *Rhagoletis*/*Drosophila* split *ca*. 65 million years ago (Grimaldi and Engel 2005). However, the absence of any identifiable orthologs of *CG9001*, *CG9002*, or *CG7573* in the *Rhagoletis* genome leads us to conclude that the serial gene duplications most likely occurred after the most recent common ancestor of *Rhagoletis* and *Drosophila*.

Although not tested in this study, *CG7573* may also be included in this group of type I prenyl proteases that are required in the male germline, possibly explaining why knockout of *CG9000*, *CG9001*, and *CG9002* does not result in complete sterility. *CG7573* is also upregulated solely in testis according to FlyAtlas, and it underexpressed in meiotic-arrest mutants (H. White-Cooper, personal communication). *CG7573* is also conserved throughout all the *Drosophila* species that have been sequenced (except *D. persimillis* where the sequence is incomplete), and it shows increased evolutionary rates when compared with *CG9000* (Table S4). Interestingly, *CG7573* may have arisen due to a retrotransposition event that precedes the diversification of the 12 sequenced *Drosophila* species: it resides on a different chromosome from the *CG9000* cluster, is intronless in 6 out of 12 *Drosophila* species, with introns only appearing in the *Drosophila* group consisting of *D. melanogaster*, *D. simulans*, *D. yakuba*, *D. erecta*, and *D. sechellia*, suggesting that the intron presence is a derived feature of the *melanogaster* subgroup and is not ancestral (Tweedie *et al.* 2009).

It is not clear what role *CG30461* may play in the male germline (or anywhere else). Although it can be expressed as an independent transcript, it bears the hallmarks of a nonprocessed pseudogene (D’Errico *et al.* 2004). Additional data supporting this hypothesis can be found in our negative selection analysis: *CG30461* demonstrates a decrease in the purifying selection that presumably maintains the specific functions of the other paralogs (Table 2). Cotranscription with *CG9002* may be due to a simple transcriptional read-through effect arising from proximity or perhaps due to something more complex, providing additional regulatory functions by lengthening a putative 3′ UTR or retarding expression of *CG9002*. Our data are inconclusive on this matter, and the evidence for distinct functional roles for pseudogenes is not abundant (but certainly not irrelevant) (Pink *et al.* 2011).

A close examination of all the stages of male germline development revealed that loss of the tandem type I prenyl protease genes in *Drosophila* appears to cause a block late in spermatogenesis. A large number of excellent studies have characterized many genes required for this stage [*e.g.* Fabrizio *et al.* (1998)]. Triple knockout males can make functional sperm but most of it accumulates at the base of the testes; also the sperm tails show signs of a surface abnormality along the length of the axoneme. Based on the methods we have applied in this report, three mechanistic explanations come to mind. First, loss of type I prenyl protease activity in the male germline may cause defects in sperm packaging and transport. This is supported by evidence of improper coiling and packing of the mature spermatids at the base if the testes in mutant males and by the reduced levels of sperm in the seminal vesicles. Also, a dramatic reduction in mature individualization complexes in mutant males might be explained by a spermatid trafficking defect that slows down or stalls the production of new individualizing cysts of spermatids.

Second, cytoplasmic remodeling requires much reorganization and polarization of the actin cytoskeleton, processes in which low molecular weight GTPases of the Rho family are famously involved (Hall 1998). Rho proteins (Rac, Cdc42, and others) maintain their stable associations with membranes via prenylation (Michaelson *et al.* 2003). *Drosophila* Rac1 is a Rho GTPase family member that has a reported male semisterile phenotype that is enhanced in the presence of a mutation in *cdi* (*center divided*), a kinase that interacts with RacGAP84C (a Rac GTPase activating protein) (Raymond *et al.* 2004). Dissected testes from these double mutants strongly resemble the phenotype of the triple knockout males reported here, which suggests that the Rac1 GTPase might well be a target of a type I prenyl protease. Interestingly, *Drosophila* has three Rac GTPases (*Rac1* or *CG2248*, *Rac2* or *CG8556*, and *Mtl* or *CG5588*). All three proteins possess motifs that would be recognized by the prenyl processing machinery (Maurer-Stroh and Eisenhauber 2005). Do any of these potential targets for type I prenyl proteases also exhibit germline-specific subfunctionalization?

A third mechanistic possibility implicates mitochondria. The axonemes of sperm tails require large numbers of mitochondria to provide ATP fuel for their construction and motility. In *Drosophila*, the mitochondria begin a remarkable process of coalescence and fusion in early spermatids, where they form a structure called a nebenkern, consisting of two intertwined giant mitochondrial derivatives (Fuller 1993). As the spermatids elongate, the two mitochondrial derivates unwrap and extend alongside the lengthening axoneme. Mitochondrial fusion is in fact mediated by a number of proteins, including a GTPase called fuzzy onions (Hales and Fuller 1997), but this GTPase has no known prenylation motifs. A recent study has demonstrated that interactions between the giant mitochondrial derivatives and the axoneme microtubule array actually drive sperm-tail elongation (Noguchi *et al.* 2011). We could not find any obvious mitochondrial abnormalities at the level of phase and fluorescence microscopy. Further ultrastructural analysis using transmission electron microscopy might well yield more conclusive findings. A mitochondrial mechanism to explain the specific defect along the lengths of the sperm tails is suggested by the punctuated localization of the Don Juan–GFP fusion protein. Don Juan is a small basic histone-like protein that localizes in haploid nuclei during chromatin condensation, and then becomes associated (via an unknown mechanism) with the mitochondria along the flagellum (Santel *et al.* 1998; Blumer *et al.* 2002). Perhaps the more punctuated Don Juan staining pattern in the prenyl protease triple knockout represents a defect along the length of the axoneme membrane, due to improper mitochondrial elongation or to defects in cytoplasmic remodeling. We favor the latter hypothesis, because an analysis of the *Drosophila* prenylated proteome reveals a large number of proteins involved with actin-based cytoskeletal reorganization and none that have any direct connection with mitochondrial function (Maurer-Stroh and Eisenhaber 2005). Of course, the two hypotheses are not mutually exclusive: mitochondrial and spermatid elongation processes are probably interdependent and involve complex driving forces.

A unifying explanation may be found in a closer examination of spermatid gigantism, an evolutionary strategy exploited by *Drosophila* species (Joly *et al.* 2008; Immler *et al.* 2011). Drosophilids are unusual in manufacturing enormous sperm—sometimes many times longer than absolute body length (Pitnick *et al.* 1995)—a strategy that sacrifices sperm number for size. This strategy is connected to sperm competition and the ability of females to select sperm for fertilization after mating (cryptic female choice) (Birkhead 1998). *Drosophila* are also unique in having proliferated their type I prenyl protease genes (at least based on current genome sequences available), and the male germline subfunctionalization is striking. Our preliminary assessment of the *Rhagoletis* genome indicates that this very closely related genus does not have multiple type I prenyl proteases, and an examination of sperm from two *Rhagoletis* species demonstrates typically small sperm, approximately 1/16 the length of *D. melanogaster* sperm (data not shown). It is tempting to speculate that increased sperm length is accompanied by a diversification of genetic functions critical for a concomitant extensive cytoskeletal remodeling.

## Supplementary Material

Supporting Information
