# Supplementary material for: Type-I Prenyl Protease Function Is Required in the Male Germline of Drosophila melanogaster
Source: G3 (Bethesda). 2012 Jun 1;2(6):629–42. doi: 10.1534/g3.112.002188 (PMC3362292; doi:10.1534/g3.112.002188)
Supplement: Supporting Information [file supp_2_6_629__index.html]

Supporting Information 

# Type-I Prenyl Protease Function Is Required in the Male Germline of *Drosophila melanogaster*

## Supporting Information for Adolphsen *et al*, 2012

**Files in this Data Supplement:**

- Supporting Information - Figures S1-S11 and Tables S1-S4 (PDF, 13.7 MB)
- Figure S1 - Clustal W alignment of sequences for the type I prenyl protease and the Drosophila paralogs (PDF, 1.4 MB)
- Figure S2 - A representative multiplex PCR result using genomic DNA from potential triple knock-out lines (PDF, 3.2 MB)
- Figure S3 - Strategy used to recover and maintain the recombinant balancer chromosome (PDF, 285 KB)
- Figure S4 - A triple knock-out (3KO) for the type I prenyl protease in Drosophila has a modest but statistically significant effect on life span in males only (PDF, 121 KB)
- Figure S5 - Clustal W alignment of CG9002 and CG30461 from the Drosophila subgroup (D.melanogaster, D. erecta, D. yakuba, D. annanasae), indicating sequence homology (PDF, 1.0 MB)
- Figure S6 - Phylogenetic analysis of the STE24 paralogs in Drosophila, including the potential non-processed pseudogene CG30461 (PDF, 254 KB)
- Figure S7 - *CG9002* and *CG30461* can be expressed independently and also together as a dicistronic unit (PDF, 1.8 MB)
- Figure S8 - PCR analysis of potential rescues (PDF, 485 KB)
- Figure S9 - The number of individualization actin complexes decreases dramatically in aged triple knock-out (3KO) males relative to their age-matched heterozygous siblings (PDF, 120 KB)
- Figure S10 - Don-Juan GFP only images of testes dissected from heterozygous (A,B,C) and homozygous (D,E,F) triple knock-out flies (PDF, 1.4 MB)
- Figure S11 - S11 Montage of Lamin Dm0 and Don-Juan (where applicable) in testes (A-C through A'''-C''' inclusive; scale bar for A panels is 40 �M; scale bar for B and C panels is 20 �M) and salivary glands (D',D'',D'''; scale bar 20 �M). (PDF, 4.3 MB)
- Table S1 - Primers used in this study (PDF, 63 KB)
- Table S2 - Penetrance and expressivity test (PDF, 71 KB)
- Table S3 - Annotation of Rhagoletis genomic sequence contigs resulting from tblastx analysis (PDF, 80 KB)
- Table S4 - Results of relative rate tests between *D. melanogaster* paralogs and *Anopheles* and *Rhagoletis STE24* (PDF, 75 KB)
