# Supplementary material for: Type-I Prenyl Protease Function Is Required in the Male Germline of Drosophila melanogaster
Source: G3 (Bethesda). 2012 Jun 1;2(6):629–42. doi: 10.1534/g3.112.002188 (PMC3362292; doi:10.1534/g3.112.002188)
Supplement: Supporting Information [file supp_2.6.629_002188SI.pdf]

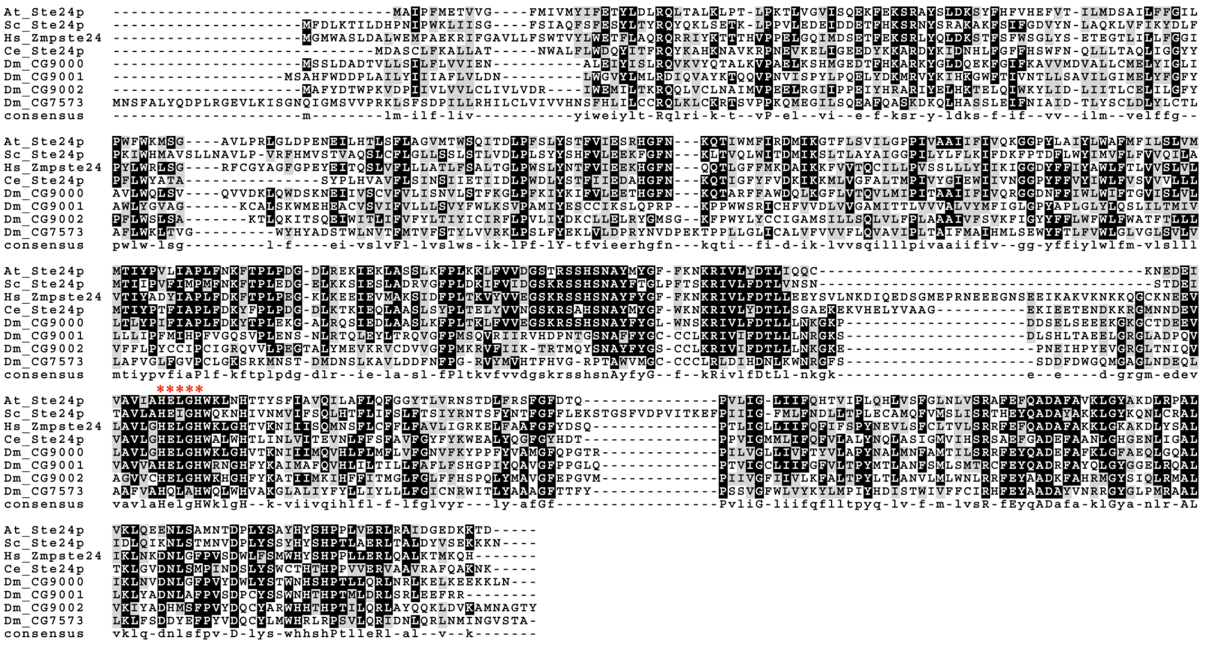

Figure S1 Clustal W alignment of sequences for the type I prenyl protease and the Drosophila paralogs. All sequences were obtained through the relevant genome project websites. The canonical Zinc ion binding site (HEXXH) that comprises the active site of the enzyme is indicated with asterisks (*). At: *Arabidopsis thaliana*; Sc: *Saccharomyces cerevisiae*; Ce: *C. elegans*; Hs: *Homo sapiens*; Dm: *Drosophila melanogaster.*

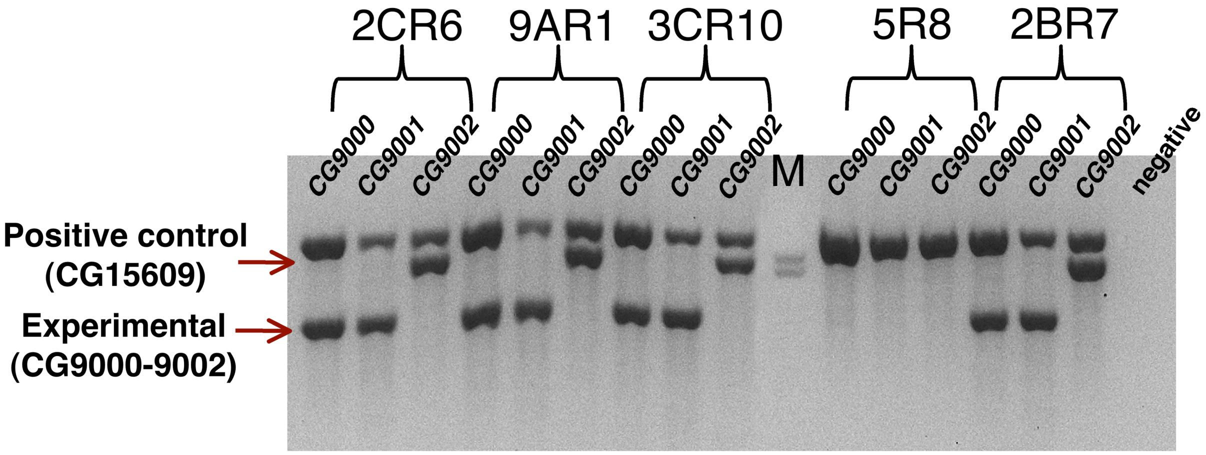

Figure S2 A representative multiplex PCR result using genomic DNA from potential triple knock-out lines. The positive control is a gene (*CG15609*) in the vicinity of the tandem *STE24* cluster, not removed by the targeted deletion. Experimental bands arise from PCR primers for *CG9000*, *CG9001* or *CG9002*. 2CR6, 9AR1, 3CR10, 5R8 and 2BR7 are all potential triple knock-out lines. All are false positives except for line 5R8, where the three tandem prenyl proteases are shown to be absent. PCR programming and conditions are described in Methods. Primer positions are depicted in Figure 1A, and sequences in Table S1.

(y)w/Y ; 3KO-w+ / CyO-3KO-w+; +/+ X *w/w ; apterous*^{Xa} */ CyO; TM6*

Captured recombinant with 3KO on balancer — *rebuilt from In(2LR)Cy to include the stronger balancer*

↓

(y)w/Y ; apterous^{Xa} */ CyO-3KO-w+; apterous*^{Xa}*/+* X *(y)w/(y)w ; apterous*^{Xa} */ CyO-3KO-w+; apterous*^{Xa}*/+*

Figure S3 Strategy used to recover and maintain the recombinant balancer chromosome. On two occasions, a balanced male was recovered that appeared to have a double dose of the wild type red eye color (*w+*) reporter that identified the triple knock-out (3KO) chromosome. In both cases, the male was virtually sterile, but when crossed to a stock bearing the reciprocal translocation *apterous*^{Xa}, it was possible to recover the *CyO* balancer bearing the triple knock out and maintain it in a stock. PCR was used to confirm the balancer had lost the three Drosophila *STE24* paralogs. Note that in this reciprocal translocation (the *apterous*^{Xa} chromosome), the region containing the three *STE24* paralogs now resides over on the third chromosome rather than the second. The original parental reciprocal translocation chromosome *apterous*^{Xa} was obtained from the Bloomington Indiana Stock center, stock #3234, where the balancer for the second is *In(2L)Cy, In(2R)Cy, Cy[1].* The instability for the *CG9000* region was unanticipated, and thus when discovered, the original Bloomington stock was rebuilt over *CyO; TM6.*

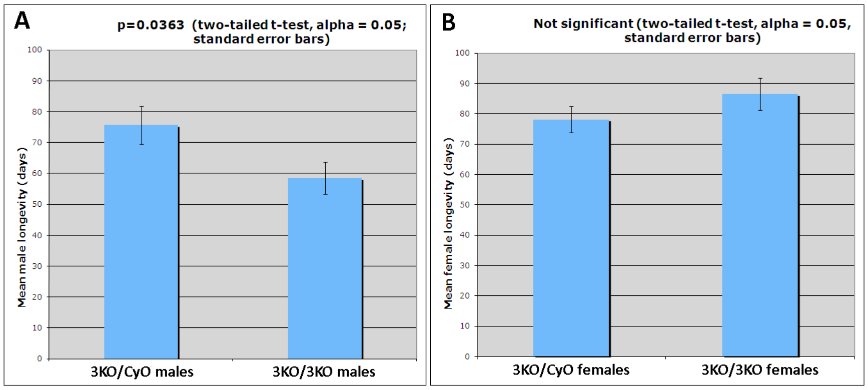

Figure S4 A triple knock-out (3KO) for the type I prenyl protease in Drosophila has a modest but statistically significant effect on life span in males only.

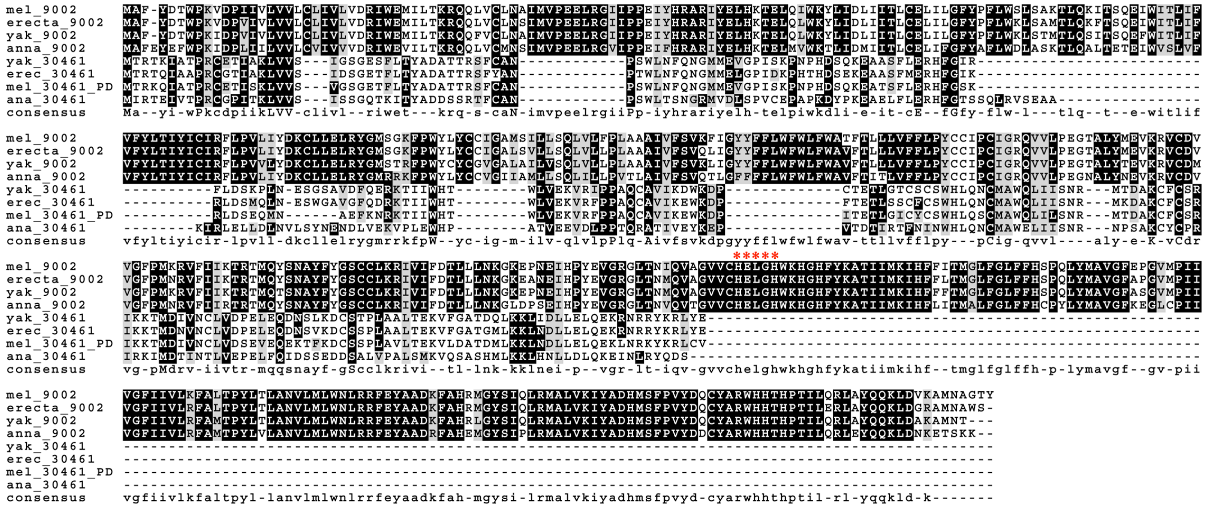

Figure S5 Clustal W alignment of *CG9002* and *CG30461* from the Drosophila subgroup (*D.melanogaster, D. erecta, D. yakuba, D. annanasae*), indicating sequence homology. Significant similarity reveals itself when *CG30461* is aligned solely with *CG9002* sequences from the most recently evolved members of the Drosophila genus. The HEXXH zinc ion binding site is indicated with asterisks (*).

Thompson, J. D., D. G. Higgins, and T. J. Gibson, 1994 Clustal W: improving the sensitivity of progressive multiple sequence alignments through sequence weighting, position specific gap penalties and weight matrix choice. Nucl. Acids Res. **22:** 4673-4680.

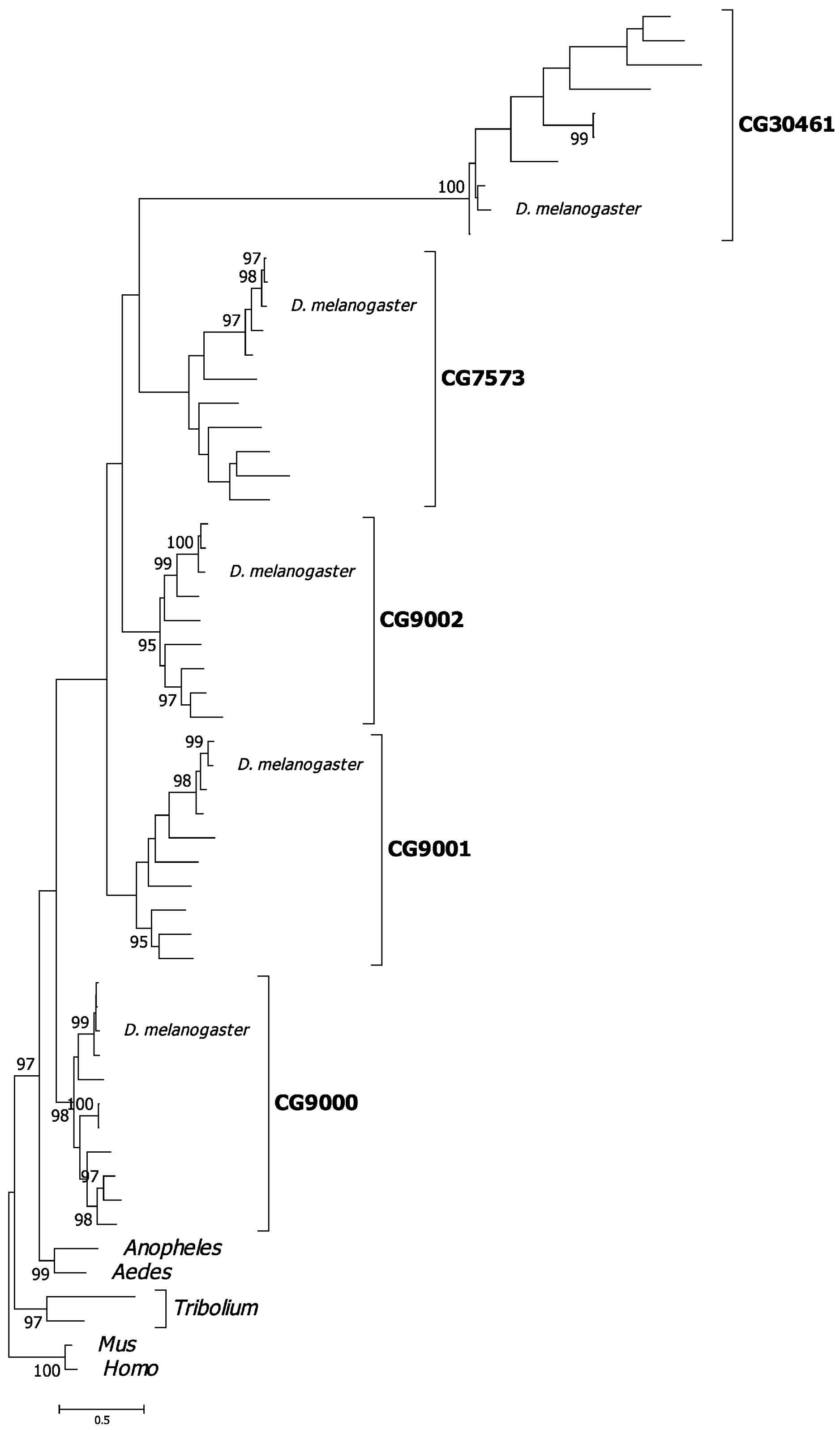
CG30461
99
100
D. melanogaster
97
98
D. melanogaster
97
CG7573
100
99
D. melanogaster
CG9002
95
97
99
D. melanogaster
98
CG9001
95
D. melanogaster
99
97
100
98
CG9000
97
98
Anopheles
99
Aedes
Tribolium
97
Mus
100
Homo
0.5

Figure S6 Phylogenetic analysis of the *STE24* paralogs in Drosophila, including the potential non-processed pseudogene *CG30461.* Maximum Likelihood phylogeny for *CG9000* and paralogs including *CG30461*. CG numbers denote the clades of Drosophila species for the respective locus. The figures above the nodes are the results from bootstrap analysis with 500 replicates (only bootstrap values >/= 95 are shown). The scale is given as substitutions per site.

Tamura, K., D. Peterson, N. Peterson, G. Stecher, M. Nei, et al., 2011 MEGA5: Molecular Evolutionary Genetics Analysis using Maximum Likelihood, Evolutionary Distance, and Maximum Parsimony Methods. Mol. Biol and Evol. **10.1093**/molbev/msr121.

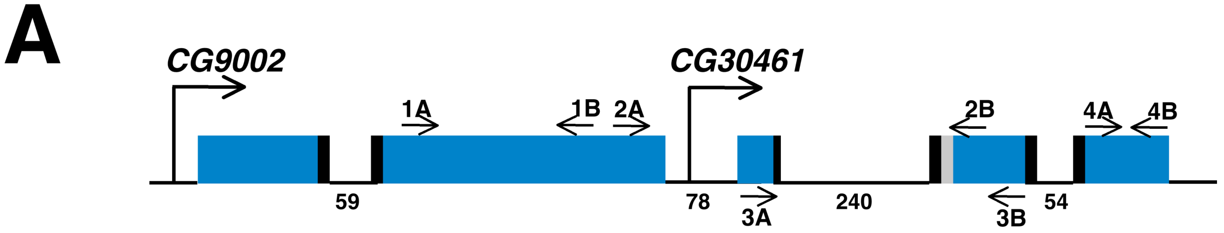

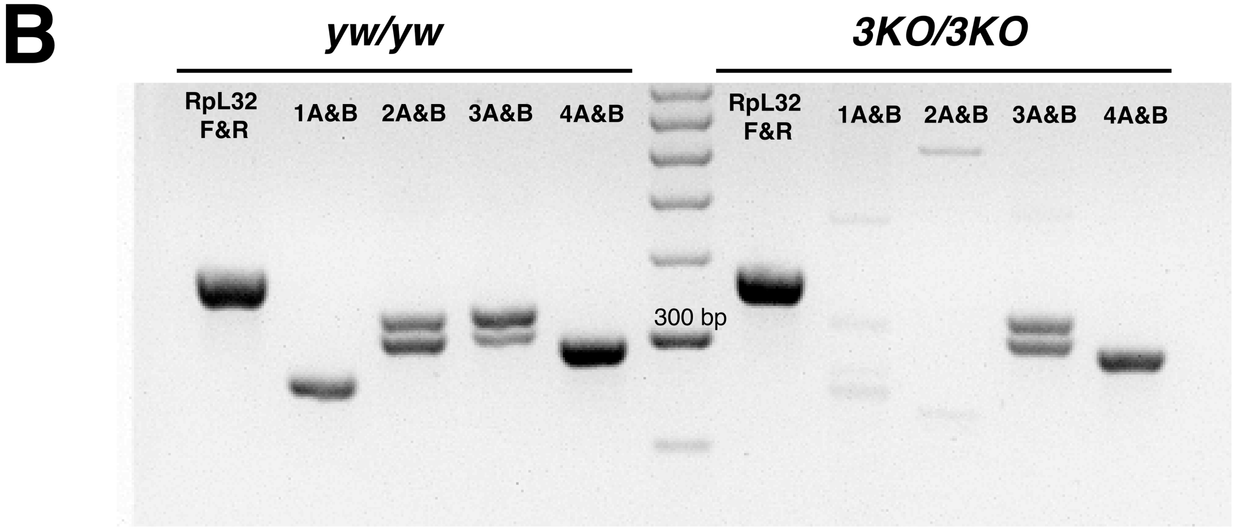

Figure S7 *CG9002* and *CG30461* can be expressed independently and also together as a dicistronic unit. (A): Diagram (not to scale) depicting the genomic structure of the region containing *CG9002* and *CG30461*. Blue boxes indicate exons, right-angle arrows indicate putative promoter regions, black bars represent splicing consensus sequences, with a grey bar indicating an alternative site in the second exon of *CG30461*. The intergenic space and all intron sizes are indicated in base pairs. Horizontal arrows indicate position of primers used to distinguish fusion from unique transcripts. (B): representative reverse transcriptase PCR result (one replicate from multiple RNA extractions) indicating existence of both fusion and unique transcripts arising from the *CG30461* locus, and the presence of multiple transcripts due to alternative splice sites in the first intron of *CG30461*. *yw* refers to a wild type stock for the prenyl protease genes; *3KO* refers to the triple knock-out genotype. *RpL32* (AKA *rp49*) was used as an external control; F & R refer to forward and reverse primers (Table S1). This experiment also shows that *CG30461* can be expressed independently of *CG9002*.

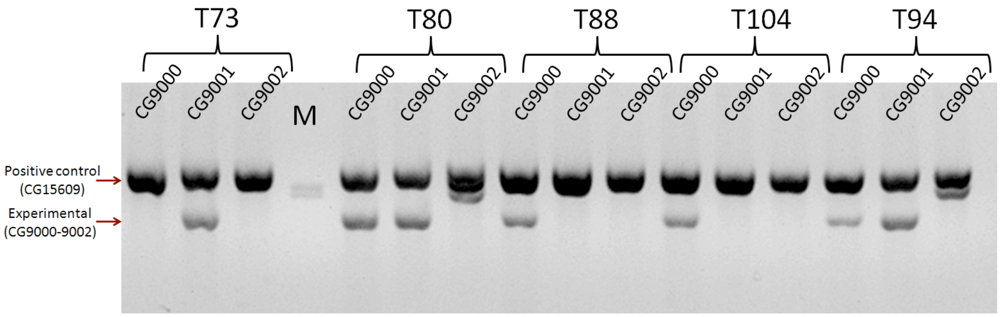

Figure S8 PCR analysis of potential rescues. Multiplex PCR was used to detect which rescue experiments represent genomic rescue from the balancer (three genes restored) and which represented rescue from the transgene (only one gene restored). For every cross, a single test male (T) was crossed to 3-4 triple knock-out females, and DNA both the parents and resulting progeny was extracted and subjected to multiplex PCR as described in the materials and methods. So in the example shown above, T80 and T94 (T = test male) are PCR results generated from DNA obtained from all parents and progeny (up to 50 flies) in which rescue has occurred due to a recombination event from the balancer in the mother's germ line (the test male was recombinant; the genes are back). T73 represents PCR results generated from DNA obtained from all parents and progeny of a potential transgenic *CG9001* rescue, and T88 and 104 are potential *CG9000* rescues.

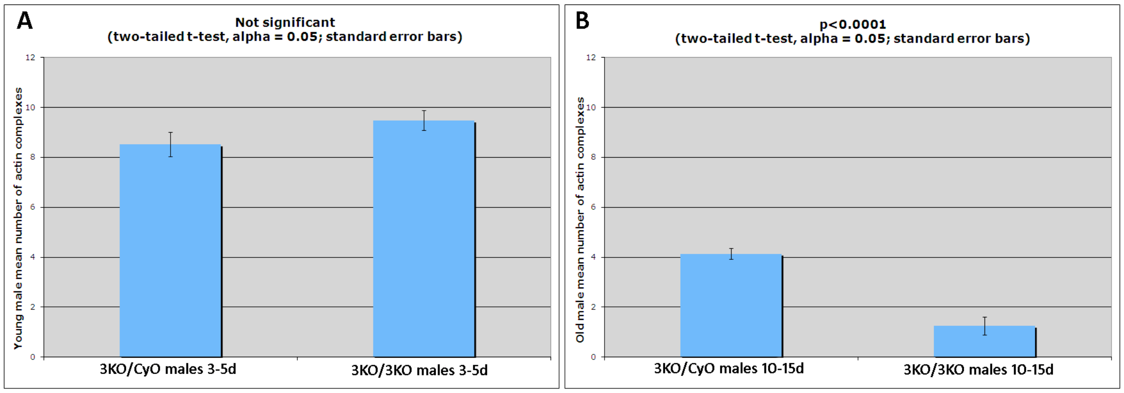

Figure S9 The number of individualization actin complexes decreases dramatically in aged triple knock-out (3KO) males relative to their age-matched heterozygous siblings.

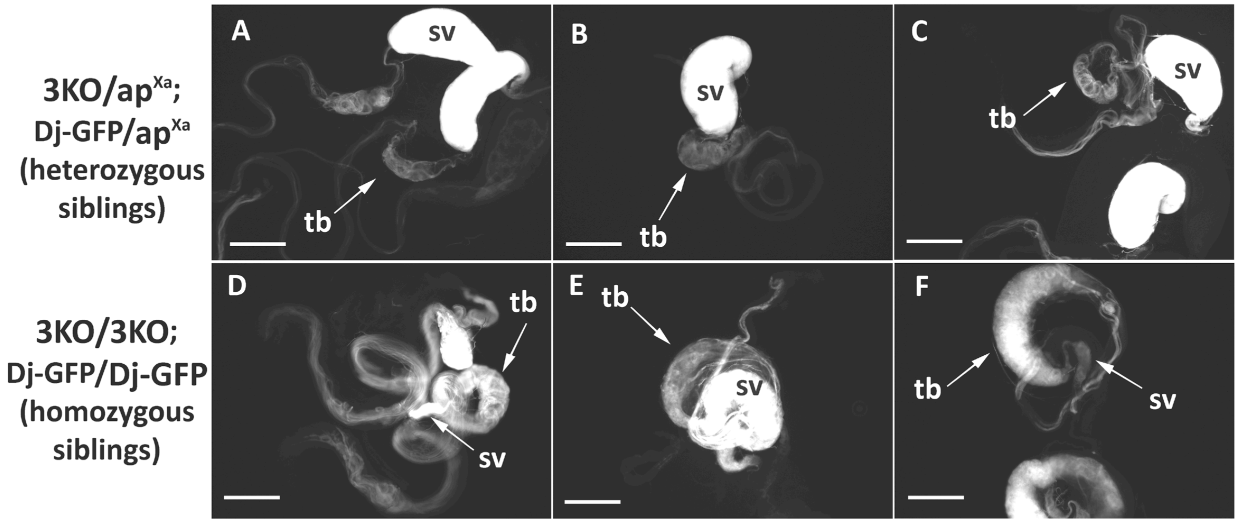

Figure S10 Don-Juan GFP only images of testes dissected from heterozygous (A,B,C) and homozygous (D,E,F) triple knock-out flies. SPOT camera settings were identical for all figures (brightness 1.1, gain 2, gamma 1). Fluorescence throughout the length of the testes corresponding to elongating individualizing spermatids appears relatively unchanged between heterozygotes and homozygotes, but there is a distinct difference in the brightness of the seminal vesicles, suggesting that one manifestation of type I prenyl protease loss is a reduced ability to move mature spermatids into the seminal vesicles. tb= testis base; sv = seminal vesicles. Scale bar is 200 μM.

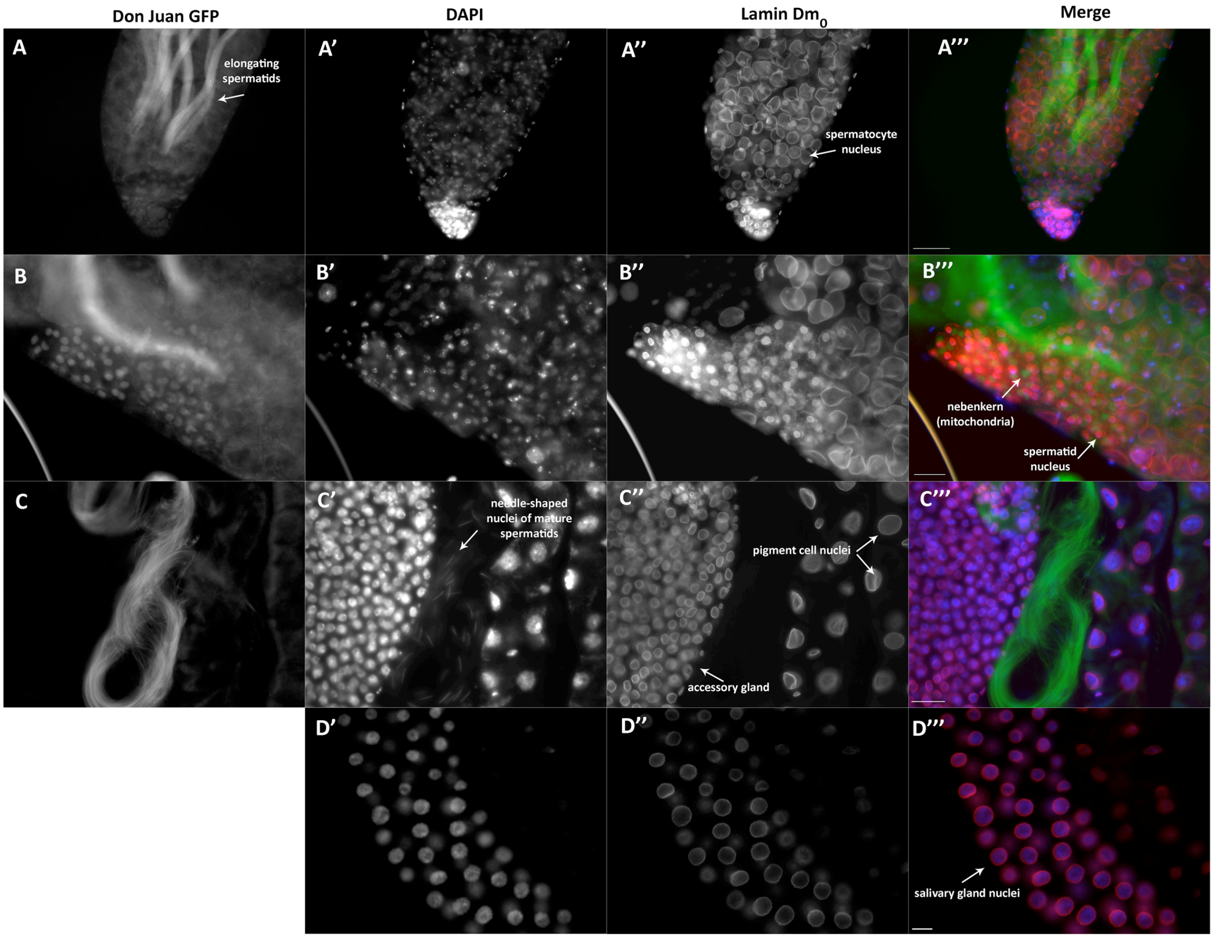

Figure S11 Montage of Lamin Dm0 and Don-Juan (where applicable) in testes (A-C through A'''-C''' inclusive; scale bar for A panels is 40 μM; scale bar for B and C panels is 20 μM) and salivary glands (D',D'',D'''; scale bar 20 μM). Normal nuclear rim staining for Lamin Dm_0 is evident in primary spermatocytes and pigment cells (present in all images) of both genotypes (wild type and triple knoc-out). Lamin Dm_0 is more diffuse in onion-stage spermatids (B: scale bar is 20μM), coincident with the coalescence of mitochondria into Nebenkern (round structures that appear like an onion in cross-section, hence the name onion stage). In part C (scale bar is 20μM), Lamin Dm_0 can only be seen localizing to the nuclei of the pigment cells that mark the sheath covering the testes. There is no trace of Lamin Dm_0 in the nuclei of mature spermatids. Normal nuclear rim staining of Lamin Dm_0 is also evident in salivary glands of both genotypes (D panels). All males are 3-4 weeks old and mated, and the montage represents the best pictures pooled from triple knock-out homozygotes and heterozygotes, as they were indistinguishable for Lamin Dm_0 localization.

Table S1 Primers used in this study

Primer name	Primer sequence
9000NotI a	ATA TAT GC GGCC GC TGT CCG TCC GTT GCG TGA G
9000SacII c	ATA TAT CCGC GG CAT GTG TGA CTT CAG TTC GGC
9000AscI b	ATA TAT GG CGCG CC GGC CTA TCA GCA GAA ACT GG
9000AvrII b	ATA TAT CCT AGG CGA GTG TCA ACG GCA GAG AAC
9000HRGSP20 (Forward for CG9000)	CGT TAT CTC GCT GGT TCT ACT C
9000HRGSP21 (Reverse for CG9000)	CAC GTA GAA TGG TGG ATA CTT G
9000HRGSP22 (Forward for CG9001)	TTA TCA GCC CCT ATC TAC CAC A
9000HRGSP23 (Reverse for CG9001)	TGA AGG TAC AAG CCC AGG GG
9000HRGSP24 (Forward for CG9002)	GGC AGG TAA GTA GTG GAT TGG T
9000HRGSP25 (Reverse for CG9002)	GTA CCC AAT GAA CTT TAC GCT G
CG6805 GSP1	CCA TGT GGG ACT GAT AAG ACA G
CG6805 GSP2	TCG ACT TTC GCT CTA CGG AA
CG15609 GSP1	GTG ATC CGA AGG TCC TAC AG
CG15609 GSP2	GTT CAC AAA GCA CGA ATG GG
Rp49 (AKARpL32) F	GAC CAT CCG CCC AGC ATA
Rp49 (AKARpL32) R	CGT TGG GGT TGG TGA GGC
9002RTF (1A in Figure S7)	CAATCATAGTGCTGGTGGTG
9002RTR (1B in Figure S7)	CAG AGG AAT GGA TAG AAG CC
30461RTF (3A in Figure S7)	CCG CTG TGA AAC TAT CTC C
30461RTR (3B in Figure S7)	ATT CCT TGA TGA CCG CAC AC
30461RT01F (4A in Figure S7)	AGC TCA GTG TGC GGT CAT C
30461RT01R (4B in Figure S7)	AGA TCA TTC AGT TTC TTC AGC A
9002FUS RTF (2A in Figure S7)	CTA CGA CCA ATG CTA TGC TC
9002FUS RTR (2B in Figure S7)	CTT CAG CCA CGA GGG ATT C

Table S2 Penetrance and expressivity test

	♂1	♂2	♂3	♂4	♂5	♂6	♂7	♂8	♂9	♂10	♂11	♂12	♂13	♂14	♂15
3KO/3KO	4	2	0	0	3	1	5	16	0	1	3	10	0	0	2
yw/yw	100+	100+	100+	100+	100+	100+	100+	100+	100+	100+	100+	100+	100+	100+	100+

The triple knock-out (3KO) genotype causes a fully penetrant but variably expressive fertility defect in males**.** Fifteen single triple knock-out males were crossed to four virgin homozygous triple knock-out sibs, and the crosses turned every three to five days and scored for adult progeny (the value in each cell of the table refers to the number of progeny). Controls consisted of age-matched wild type (*yw)* individual males crossed to four virgin wild type (*yw)* females and turned every three to five days, and scoring stopped after progeny numbers exceeded 100.

Table S3 Annotation of Rhagoletis genomic sequence contigs resulting from tblastx analysis

First four blastx hits ordered from most to least similar	Max identity %	E-value
***Rhagoletis* contig 1 *CG9000* search**		
CG9000	79.53	0
CG9001	40.29	2.00E-88
CG9002	37.43	6.00E-78
CG7573	26.2	6.00E-33
***Rhagoletis* contig 2 *CG9000* search**		
CG9000	56.72	3.00E-23
CG9001	31.82	3.00E-07
CG9002	31.58	8.00E-07
small wing	29.17	5.00E-04
***Rhagoletis* contig *CG9001* search**		
CG9000	83.42	1.00E-124
CG9001	52.38	6.00E-73
CG9002	46.7	3.00E-61
CG7573	31.22	9.00E-29
***Rhagoletis* contig 1 *CG9002* search**		
CG9000	78.5	3.00E-60
CG9001	51.89	2.00E-40
CG9002	45.87	2.00E-32
CG7573	34.31	2.00E-15
***Rhagoletis* contig 2 *CG9002* search**		
CG9000	88.42	5.00E-59
CG9001	52.33	2.00E-28
CG9002	48.89	3.00E-25
CG7573	29.63	2.00E-11
***Rhagoletis* contig 2 *CG4852* search**		
CG4852	79.27	7.00E-46
alpha-spectrin	42.11	0.001
rigor mortis	34.78	0.003

The Rhagoletis contigs that served as queries are in bold with the best blast hits for each query listed below. Each Rhagoletis contig was assembled from genomic reads that had been identified by a blast search as being similar to the listed *D. melanogaster* locus. Some searches of the Rhagoletis genome with a single Drosophila locus resulted in 2 separate non-overlapping contigs. Note that no contig in the Rhagoletis genome could be constructed from a search with Drosophila *CG7573* due to the absence of any homologous sequences. See Materials and Methods for further details.

Table S4 Results of relative rate tests between *D. melanogaster* paralogs and *Anopheles and Rhagoletis STE24*.

Taxon 1	Taxon 2	p-value
Anopheles *STE24* (108)	*D. mel. CG9000* (112)	0.787
Rhagoletis *STE24* (133)	*D. mel. CG9000* (141)	0.629
D. mel. CG9000 (94)	*D. mel. CG9001* (209)	< 0.00001*
D. mel. CG9000 (96)	*D. mel. CG9002* (194)	< 0.00001*
D. mel. CG9000 (100)	*D. mel. CG7573* (234)	< 0.00001*
D. mel. CG9000 (64)	*D. mel. CG30461* (227)	< 0.00001*
D. mel. CG9001 (137)	*D. mel. CG9002* (120)	0.289
D. mel. CG9001 (139)	*D. mel. CG7573* (157)	0.296
D. mel. CG9001 (85)	*D. mel. CG30461* (166)	< 0.00001*
D. mel. CG9002 (116)	*D. mel. CG7573* (155)	0.018
D. mel. CG9002 (87)	*D. mel. CG30461* (192)	< 0.00001*
D. mel. CG7573 (91)	*D. mel. CG30461* (166)	< 0.00001*

* = significant rate differences after Benjamini-Hochberg correction.
Homo sapiens served as the outgroup in all tests. The number of unique nucleotide differences for each taxon is listed in brackets.
