# Supplementary material for: Type-I Prenyl Protease Function Is Required in the Male Germline of Drosophila melanogaster
Source: G3 (Bethesda). 2012 Jun 1;2(6):629–42. doi: 10.1534/g3.112.002188 (PMC3362292; doi:10.1534/g3.112.002188)
Supplement: Supporting Information [file supp_2.6.629_FigureS2.pdf]

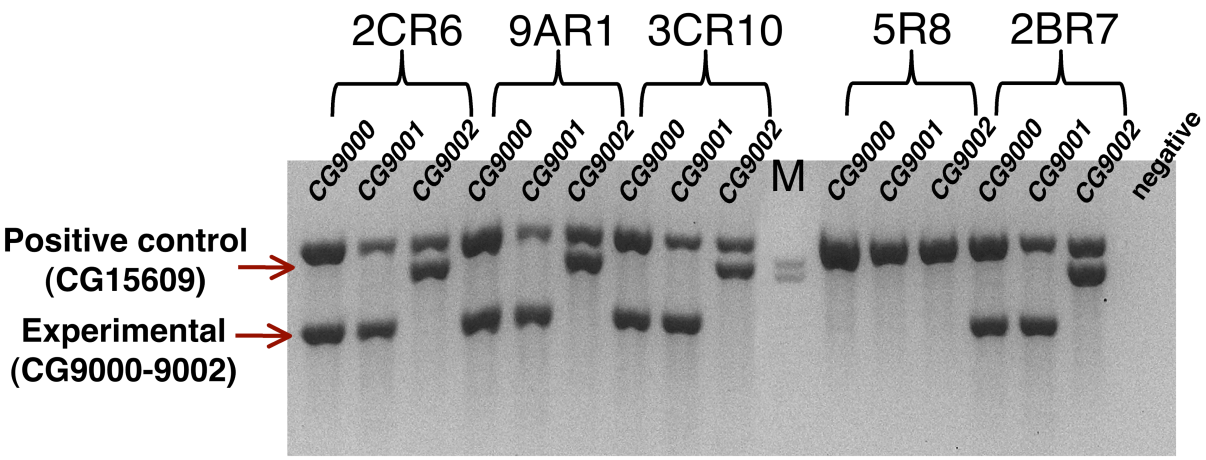

Figure S2 A representative multiplex PCR result using genomic DNA from potential triple knock-out lines. The positive control is a gene (*CG15609*) in the vicinity of the tandem *STE24* cluster, not removed by the targeted deletion. Experimental bands arise from PCR primers for *CG9000*, *CG9001* or *CG9002*. 2CR6, 9AR1, 3CR10, 5R8 and 2BR7 are all potential triple knock-out lines. All are false positives except for line 5R8, where the three tandem prenyl proteases are shown to be absent. PCR programming and conditions are described in Methods. Primer positions are depicted in Figure 1A, and sequences in Table S1.
