# Supplementary material for: Type-I Prenyl Protease Function Is Required in the Male Germline of Drosophila melanogaster
Source: G3 (Bethesda). 2012 Jun 1;2(6):629–42. doi: 10.1534/g3.112.002188 (PMC3362292; doi:10.1534/g3.112.002188)
Supplement: Supporting Information [file supp_2.6.629_FigureS3.pdf]

(y)w/Y ; 3KO-w+ / CyO-3KO-w+; +/+ X *w/w ; apterous^{Xa} / CyO; TM6*

Captured recombinant with 3KO on balancer — *rebuilt from In(2LR)Cy to include the stronger balancer*

↓

(y)w/Y ; apterous^{Xa} / CyO-3KO-w+; apterous^{Xa}/+ X *(y)w/(y)w ; apterous^{Xa} / CyO-3KO-w+; apterous^{Xa}/+*

Figure S3 Strategy used to recover and maintain the recombinant balancer chromosome. On two occasions, a balanced male was recovered that appeared to have a double dose of the wild type red eye color (*w+*) reporter that identified the triple knock-out (3KO) chromosome. In both cases, the male was virtually sterile, but when crossed to a stock bearing the reciprocal translocation *apterous*^{Xa}, it was possible to recover the *CyO* balancer bearing the triple knock out and maintain it in a stock. PCR was used to confirm the balancer had lost the three Drosophila *STE24* paralogs. Note that in this reciprocal translocation (the *apterous*^{Xa} chromosome), the region containing the three *STE24* paralogs now resides over on the third chromosome rather than the second. The original parental reciprocal translocation chromosome *apterous*^{Xa} was obtained from the Bloomington Indiana Stock center, stock #3234, where the balancer for the second is *In(2L)Cy, In(2R)Cy, Cy[1].* The instability for the *CG9000* region was unanticipated, and thus when discovered, the original Bloomington stock was rebuilt over *CyO; TM6.*
