# Supplementary material for: Type-I Prenyl Protease Function Is Required in the Male Germline of Drosophila melanogaster
Source: G3 (Bethesda). 2012 Jun 1;2(6):629–42. doi: 10.1534/g3.112.002188 (PMC3362292; doi:10.1534/g3.112.002188)
Supplement: Supporting Information [file supp_2.6.629_FigureS5.pdf]

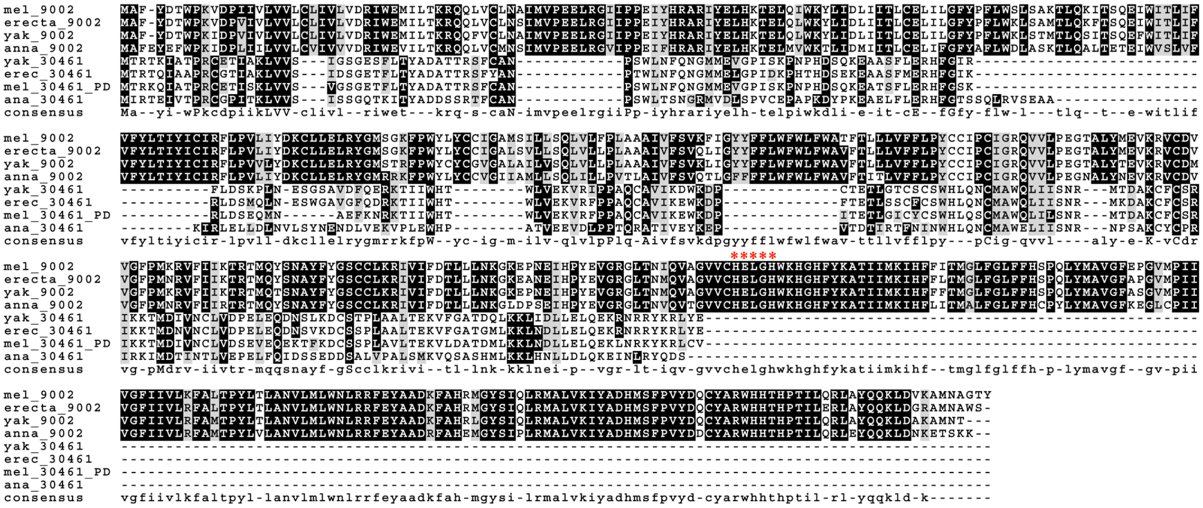

**Figure S5** Clustal W alignment of *CG9002* and *CG30461* from the Drosophila subgroup (*D.melanogaster, D. erecta, D. yakuba, D. annanasae*), indicating sequence homology. Significant similarity reveals itself when *CG30461* is aligned solely with *CG9002* sequences from the most recently evolved members of the Drosophila genus. The HEXXH zinc ion binding site is indicated with asterisks (*).

Thompson, J. D., D. G. Higgins, and T. J. Gibson, 1994 Clustal W: improving the sensitivity of progressive multiple sequence alignments through sequence weighting, position specific gap penalties and weight matrix choice. Nucl. Acids Res. **22:** 4673-4680.
