# Supplementary material for: Type-I Prenyl Protease Function Is Required in the Male Germline of Drosophila melanogaster
Source: G3 (Bethesda). 2012 Jun 1;2(6):629–42. doi: 10.1534/g3.112.002188 (PMC3362292; doi:10.1534/g3.112.002188)
Supplement: Supporting Information [file supp_2.6.629_FigureS6.pdf]

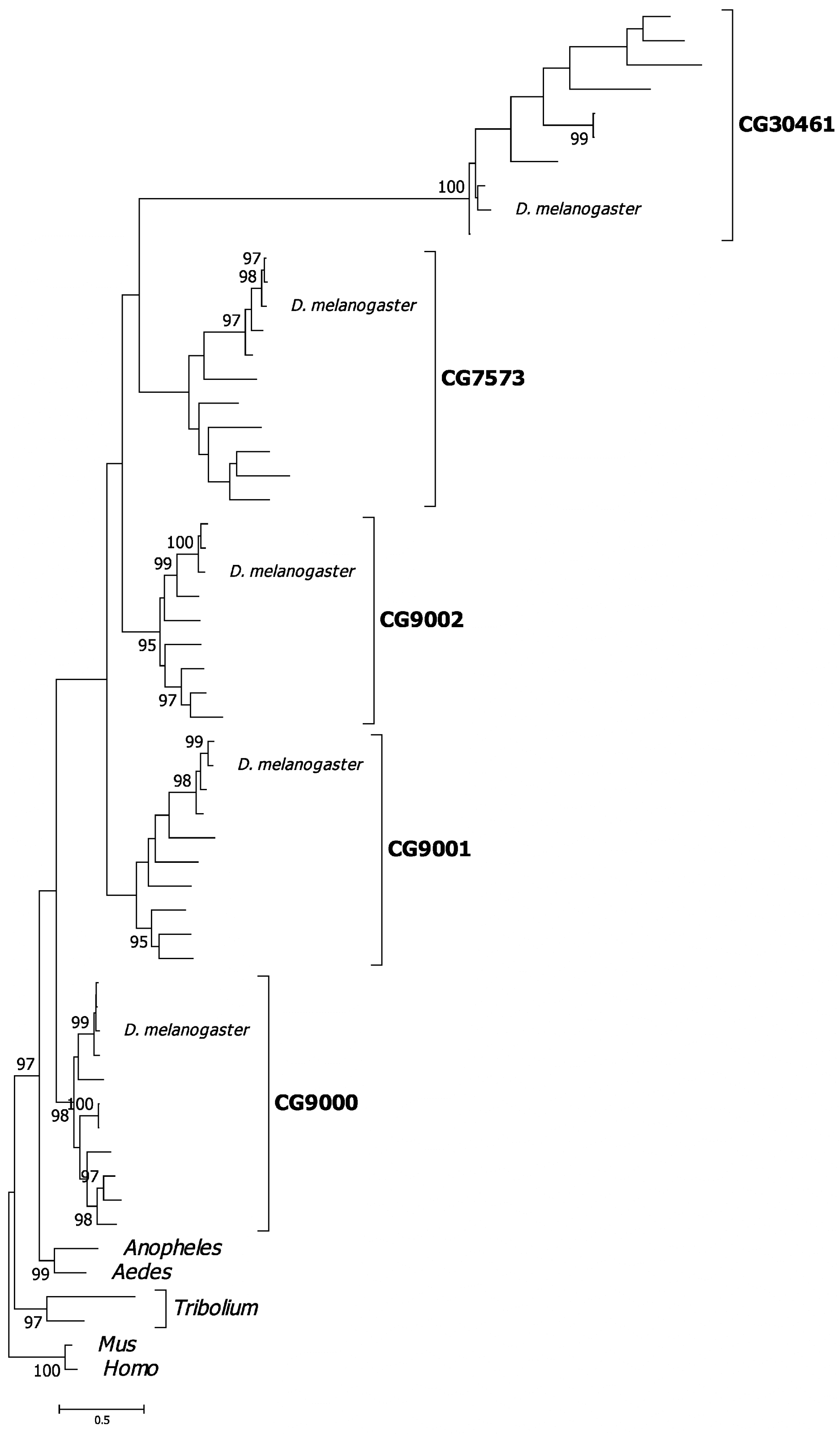
CG30461
99
100
D. melanogaster
97
98
D. melanogaster
97
CG7573
100
99
D. melanogaster
CG9002
95
97
99
D. melanogaster
98
CG9001
95
99
D. melanogaster
97
100
98
CG9000
97
98
Anopheles
99
Aedes
Tribolium
97
Mus
100
Homo
0.5

Figure S6 Phylogenetic analysis of the *STE24* paralogs in Drosophila, including the potential non-processed pseudogene *CG30461.* Maximum Likelihood phylogeny for *CG9000* and paralogs including *CG30461*. CG numbers denote the clades of Drosophila species for the respective locus. The figures above the nodes are the results from bootstrap analysis with 500 replicates (only bootstrap values >/= 95 are shown). The scale is given as substitutions per site.

Tamura, K., D. Peterson, N. Peterson, G. Stecher, M. Nei, et al., 2011 MEGA5: Molecular Evolutionary Genetics Analysis using Maximum Likelihood, Evolutionary Distance, and Maximum Parsimony Methods. Mol. Biol and Evol. **10.1093**/molbev/msr121.
