# Supplementary material for: Type-I Prenyl Protease Function Is Required in the Male Germline of Drosophila melanogaster
Source: G3 (Bethesda). 2012 Jun 1;2(6):629–42. doi: 10.1534/g3.112.002188 (PMC3362292; doi:10.1534/g3.112.002188)
Supplement: Supporting Information [file supp_2.6.629_FigureS7.pdf]

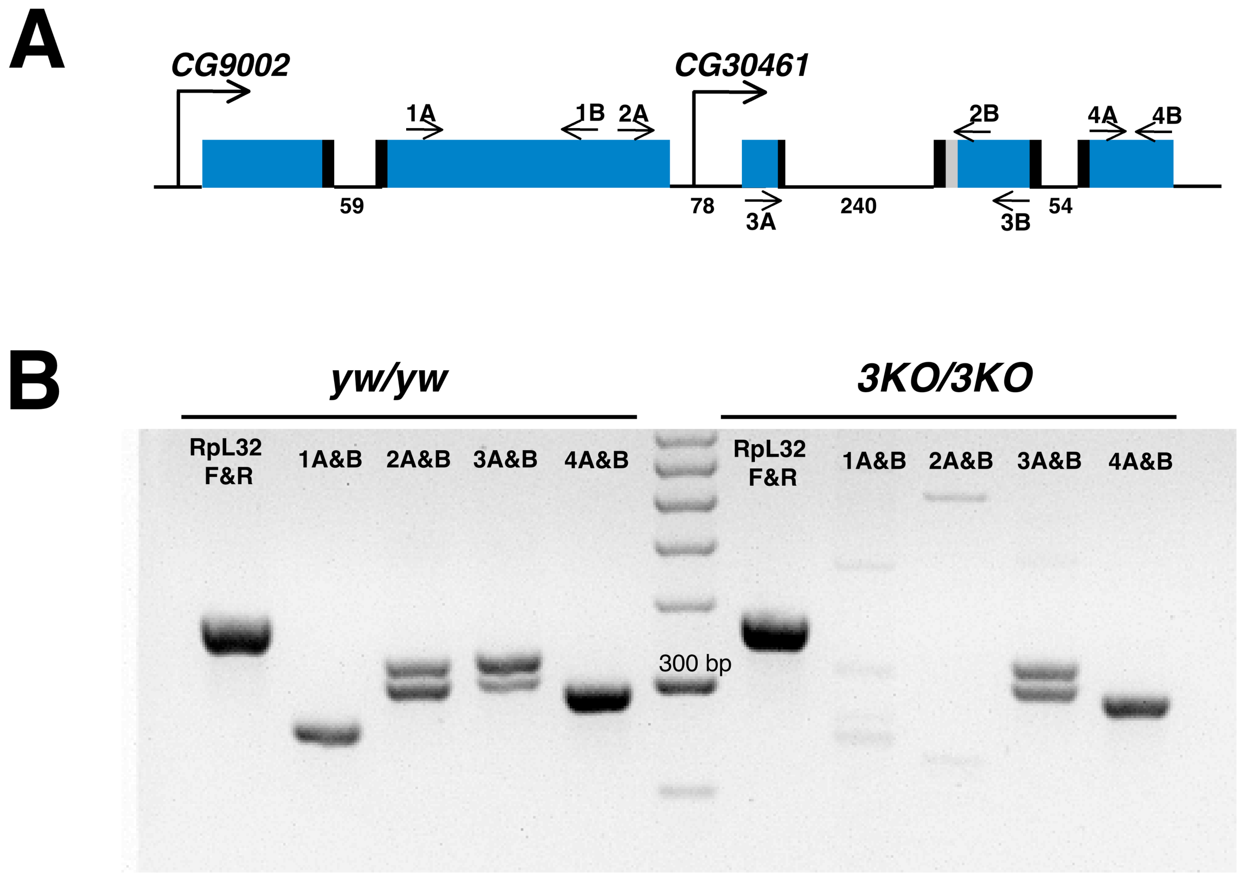

Figure S7 *CG9002* and *CG30461* can be expressed independently and also together as a dicistronic unit. (A): Diagram (not to scale) depicting the genomic structure of the region containing *CG9002* and *CG30461*. Blue boxes indicate exons, right-angle arrows indicate putative promoter regions, black bars represent splicing consensus sequences, with a grey bar indicating an alternative site in the second exon of *CG30461*. The intergenic space and all intron sizes are indicated in base pairs. Horizontal arrows indicate position of primers used to distinguish fusion from unique transcripts. (B): representative reverse transcriptase PCR result (one replicate from multiple RNA extractions) indicating existence of both fusion and unique transcripts arising from the *CG30461* locus, and the presence of multiple transcripts due to alternative splice sites in the first intron of *CG30461*. *yw* refers to a wild type stock for the prenyl protease genes; *3KO* refers to the triple knock-out genotype. *RpL32* (AKA *rp49*) was used as an external control; F & R refer to forward and reverse primers (Table S1). This experiment also shows that *CG30461* can be expressed independently of *CG9002*.
