# Supplementary material for: Type-I Prenyl Protease Function Is Required in the Male Germline of Drosophila melanogaster
Source: G3 (Bethesda). 2012 Jun 1;2(6):629–42. doi: 10.1534/g3.112.002188 (PMC3362292; doi:10.1534/g3.112.002188)
Supplement: Supporting Information [file supp_2.6.629_FigureS8.pdf]

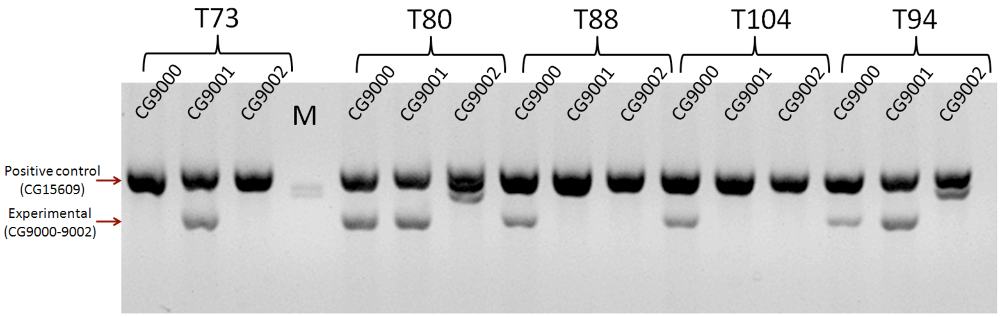

Figure S8 PCR analysis of potential rescues. Multiplex PCR was used to detect which rescue experiments represent genomic rescue from the balancer (three genes restored) and which represented rescue from the transgene (only one gene restored). For every cross, a single test male (T) was crossed to 3-4 triple knock-out females, and DNA both the parents and resulting progeny was extracted and subjected to multiplex PCR as described in the materials and methods. So in the example shown above, T80 and T94 (T = test male) are PCR results generated from DNA obtained from all parents and progeny (up to 50 flies) in which rescue has occurred due to a recombination event from the balancer in the mother's germ line (the test male was recombinant; the genes are back). T73 represents PCR results generated from DNA obtained from all parents and progeny of a potential transgenic *CG9001* rescue, and T88 and 104 are potential *CG9000* rescues.
