# Supplementary material for: Type-I Prenyl Protease Function Is Required in the Male Germline of Drosophila melanogaster
Source: G3 (Bethesda). 2012 Jun 1;2(6):629–42. doi: 10.1534/g3.112.002188 (PMC3362292; doi:10.1534/g3.112.002188)
Supplement: Supporting Information [file supp_2.6.629_FigureS9.pdf]

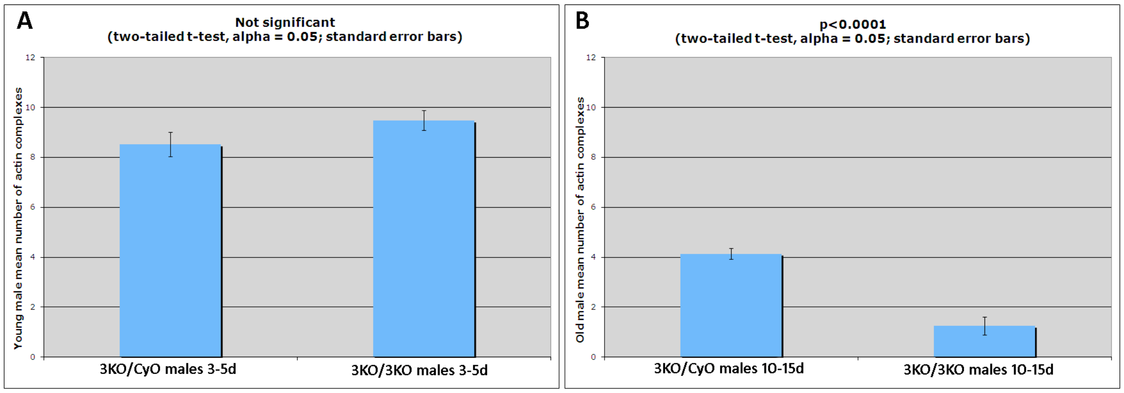

Figure S9 The number of individualization actin complexes decreases dramatically in aged triple knock-out (3KO) males relative to their age-matched heterozygous siblings.
