# Supplementary material for: Type-I Prenyl Protease Function Is Required in the Male Germline of Drosophila melanogaster
Source: G3 (Bethesda). 2012 Jun 1;2(6):629–42. doi: 10.1534/g3.112.002188 (PMC3362292; doi:10.1534/g3.112.002188)
Supplement: Supporting Information [file supp_2.6.629_FigureS10.pdf]

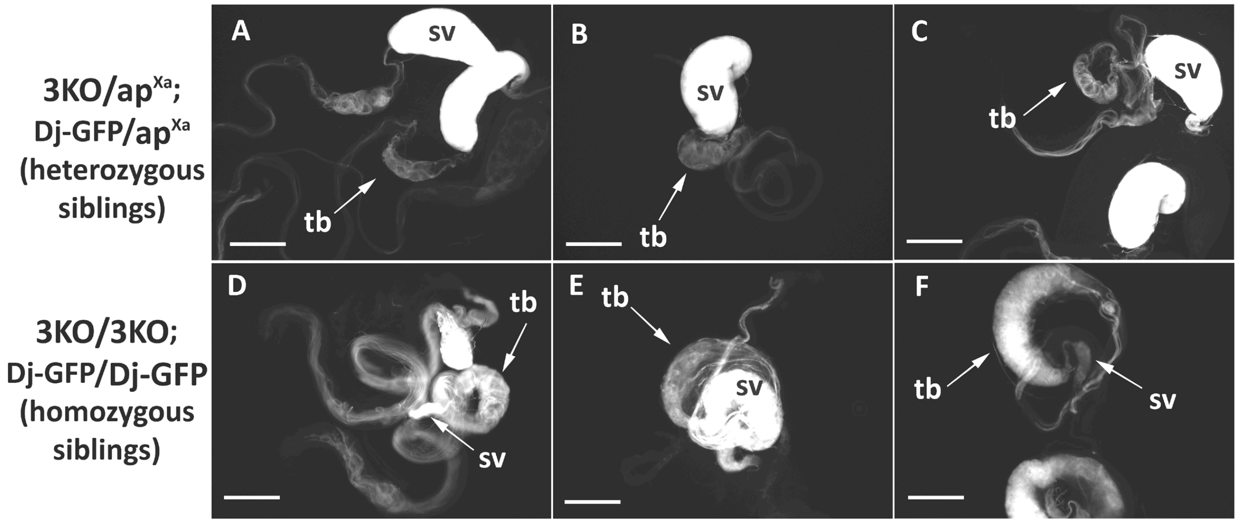

Figure S10 Don-Juan GFP only images of testes dissected from heterozygous (A,B,C) and homozygous (D,E,F) triple knock-out flies. SPOT camera settings were identical for all figures (brightness 1.1, gain 2, gamma 1). Fluorescence throughout the length of the testes corresponding to elongating individualizing spermatids appears relatively unchanged between heterozygotes and homozygotes, but there is a distinct difference in the brightness of the seminal vesicles, suggesting that one manifestation of type I prenyl protease loss is a reduced ability to move mature spermatids into the seminal vesicles. tb= testis base; sv = seminal vesicles. Scale bar is 200 μM.
