# Supplementary material for: Type-I Prenyl Protease Function Is Required in the Male Germline of Drosophila melanogaster
Source: G3 (Bethesda). 2012 Jun 1;2(6):629–42. doi: 10.1534/g3.112.002188 (PMC3362292; doi:10.1534/g3.112.002188)
Supplement: Supporting Information [file supp_2.6.629_FigureS11.pdf]

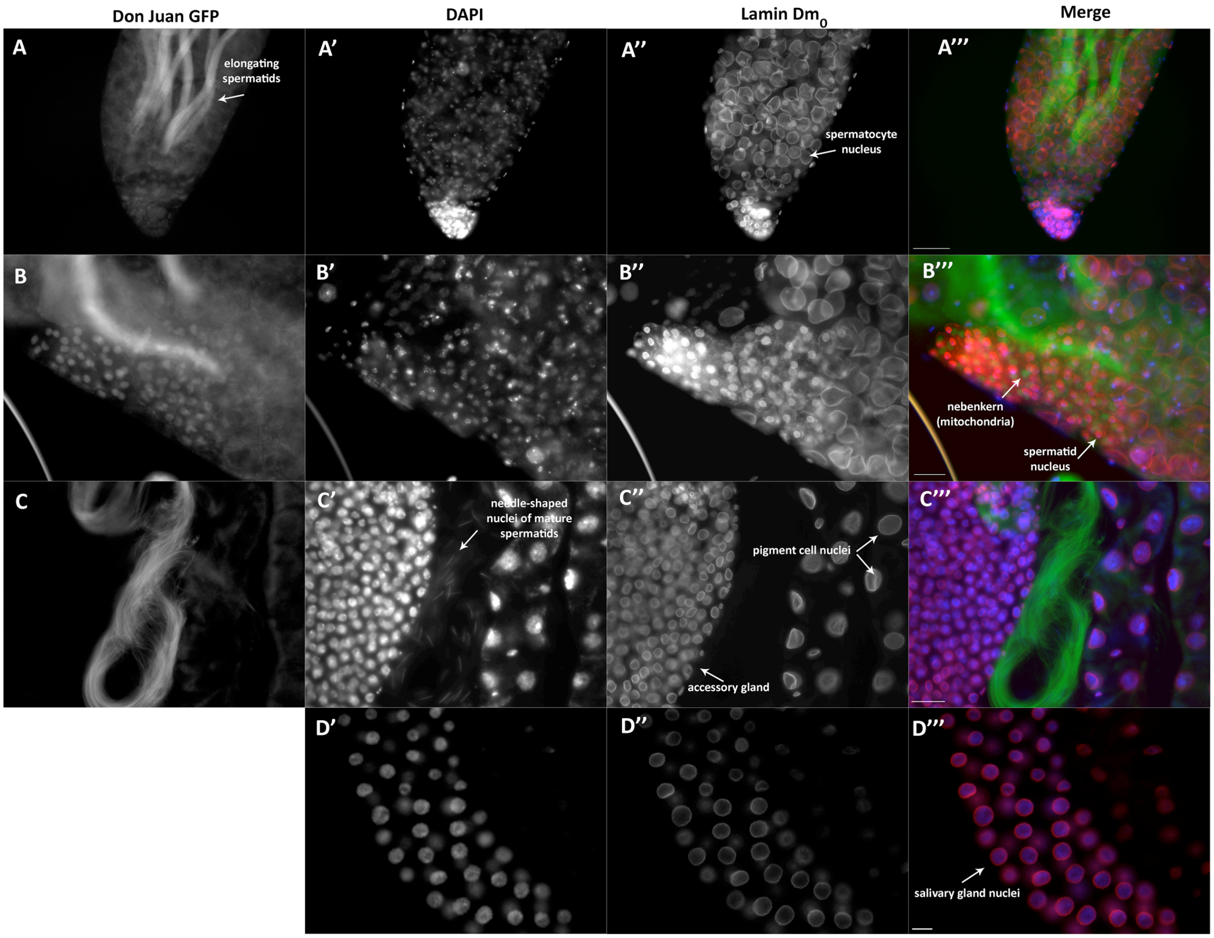

Figure S11 Montage of Lamin Dm0 and Don-Juan (where applicable) in testes (A-C through A'''-C''' inclusive; scale bar for A panels is 40 μM; scale bar for B and C panels is 20 μM) and salivary glands (D',D'',D'''; scale bar 20 μM). Normal nuclear rim staining for Lamin Dm_0 is evident in primary spermatocytes and pigment cells (present in all images) of both genotypes (wild type and triple knoc-out). Lamin Dm_0 is more diffuse in onion-stage spermatids (B: scale bar is 20μM), coincident with the coalescence of mitochondria into Nebenkern (round structures that appear like an onion in cross-section, hence the name onion stage). In part C (scale bar is 20μM), Lamin Dm_0 can only be seen localizing to the nuclei of the pigment cells that mark the sheath covering the testes. There is no trace of Lamin Dm_0 in the nuclei of mature spermatids. Normal nuclear rim staining of Lamin Dm_0 is also evident in salivary glands of both genotypes (D panels). All males are 3-4 weeks old and mated, and the montage represents the best pictures pooled from triple knock-out homozygotes and heterozygotes, as they were indistinguishable for Lamin Dm_0 localization.
