# Supplementary material for: Type-I Prenyl Protease Function Is Required in the Male Germline of Drosophila melanogaster
Source: G3 (Bethesda). 2012 Jun 1;2(6):629–42. doi: 10.1534/g3.112.002188 (PMC3362292; doi:10.1534/g3.112.002188)
Supplement: Supporting Information [file supp_2.6.629_TableS1.pdf]

Table S1 Primers used in this study

Primer name	Primer sequence
9000NotI a	ATA TAT GC GGCC GC TGT CCG TCC GTT GCG TGA G
9000SacII c	ATA TAT CCGC GG CAT GTG TGA CTT CAG TTC GGC
9000AscI b	ATA TAT GG CGCG CC GGC CTA TCA GCA GAA ACT GG
9000AvrII b	ATA TAT CCT AGG CGA GTG TCA ACG GCA GAG AAC
9000HRGSP20 (Forward for CG9000)	CGT TAT CTC GCT GGT TCT ACT C
9000HRGSP21 (Reverse for CG9000)	CAC GTA GAA TGG TGG ATA CTT G
9000HRGSP22 (Forward for CG9001)	TTA TCA GCC CCT ATC TAC CAC A
9000HRGSP23 (Reverse for CG9001)	TGA AGG TAC AAG CCC AGG GG
9000HRGSP24 (Forward for CG9002)	GGC AGG TAA GTA GTG GAT TGG T
9000HRGSP25 (Reverse for CG9002)	GTA CCC AAT GAA CTT TAC GCT G
CG6805 GSP1	CCA TGT GGG ACT GAT AAG ACA G
CG6805 GSP2	TCG ACT TTC GCT CTA CGG AA
CG15609 GSP1	GTG ATC CGA AGG TCC TAC AG
CG15609 GSP2	GTT CAC AAA GCA CGA ATG GG
Rp49 (AKARpL32) F	GAC CAT CCG CCC AGC ATA
Rp49 (AKARpL32) R	CGT TGG GGT TGG TGA GGC
9002RTF (1A in Figure S7)	CAATCATAGTGCTGGTGGTG
9002RTR (1B in Figure S7)	CAG AGG AAT GGA TAG AAG CC
30461RTF (3A in Figure S7)	CCG CTG TGA AAC TAT CTC C
30461RTR (3B in Figure S7)	ATT CCT TGA TGA CCG CAC AC
30461RT01F (4A in Figure S7)	AGC TCA GTG TGC GGT CAT C
30461RT01R (4B in Figure S7)	AGA TCA TTC AGT TTC TTC AGC A
9002FUS RTF (2A in Figure S7)	CTA CGA CCA ATG CTA TGC TC
9002FUS RTR (2B in Figure S7)	CTT CAG CCA CGA GGG ATT C
