# Supplementary material for: Type-I Prenyl Protease Function Is Required in the Male Germline of Drosophila melanogaster
Source: G3 (Bethesda). 2012 Jun 1;2(6):629–42. doi: 10.1534/g3.112.002188 (PMC3362292; doi:10.1534/g3.112.002188)
Supplement: Supporting Information [file supp_2.6.629_FigureS1.pdf]

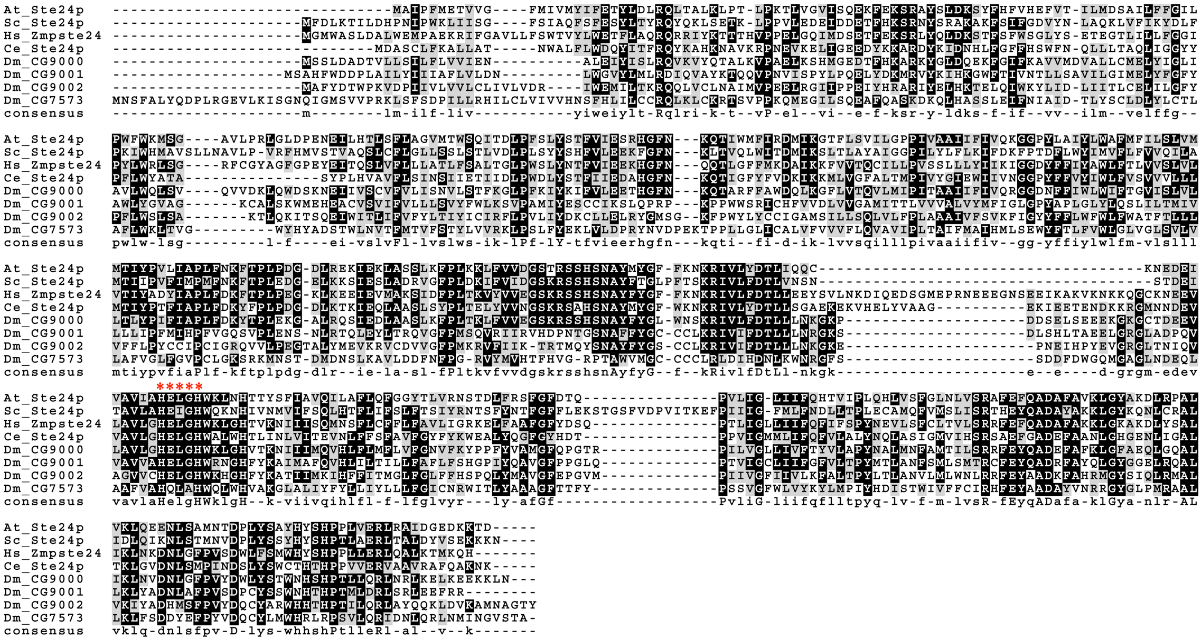

Figure S1 Clustal W alignment of sequences for the type I prenyl protease and the Drosophila paralogs. All sequences were obtained through the relevant genome project websites. The canonical Zinc ion binding site (HEXXH) that comprises the active site of the enzyme is indicated with asterisks (*). At: *Arabidopsis thaliana*; Sc: *Saccharomyces cerevisiae*; Ce: *C. elegans*; Hs: *Homo sapiens*; Dm: *Drosophila melanogaster.*
