# Supplementary material for: Type-I Prenyl Protease Function Is Required in the Male Germline of Drosophila melanogaster
Source: G3 (Bethesda). 2012 Jun 1;2(6):629–42. doi: 10.1534/g3.112.002188 (PMC3362292; doi:10.1534/g3.112.002188)
Supplement: Supporting Information [file supp_2.6.629_TableS3.pdf]

Table S3 Annotation of Rhagoletis genomic sequence contigs resulting from tblastx analysis

First four blastx hits ordered from most to least similar	Max identity %	E-value
***Rhagoletis* contig 1 *CG9000* search**		
CG9000	79.53	0
CG9001	40.29	2.00E-88
CG9002	37.43	6.00E-78
CG7573	26.2	6.00E-33
***Rhagoletis* contig 2 *CG9000* search**		
CG9000	56.72	3.00E-23
CG9001	31.82	3.00E-07
CG9002	31.58	8.00E-07
small wing	29.17	5.00E-04
***Rhagoletis* contig *CG9001* search**		
CG9000	83.42	1.00E-124
CG9001	52.38	6.00E-73
CG9002	46.7	3.00E-61
CG7573	31.22	9.00E-29
***Rhagoletis* contig 1 *CG9002* search**		
CG9000	78.5	3.00E-60
CG9001	51.89	2.00E-40
CG9002	45.87	2.00E-32
CG7573	34.31	2.00E-15
***Rhagoletis* contig 2 *CG9002* search**		
CG9000	88.42	5.00E-59
CG9001	52.33	2.00E-28
CG9002	48.89	3.00E-25
CG7573	29.63	2.00E-11
***Rhagoletis* contig 2 *CG4852* search**		
CG4852	79.27	7.00E-46
alpha-spectrin	42.11	0.001
rigor mortis	34.78	0.003

The Rhagoletis contigs that served as queries are in bold with the best blast hits for each query listed below. Each Rhagoletis contig was assembled from genomic reads that had been identified by a blast search as being similar to the listed *D. melanogaster* locus. Some searches of the Rhagoletis genome with a single Drosophila locus resulted in 2 separate non-overlapping contigs. Note that no contig in the Rhagoletis genome could be constructed from a search with Drosophila *CG7573* due to the absence of any homologous sequences. See Materials and Methods for further details.
