# Supplementary material for: Type-I Prenyl Protease Function Is Required in the Male Germline of Drosophila melanogaster
Source: G3 (Bethesda). 2012 Jun 1;2(6):629–42. doi: 10.1534/g3.112.002188 (PMC3362292; doi:10.1534/g3.112.002188)
Supplement: Supporting Information [file supp_2.6.629_TableS4.pdf]

Table S4 Results of relative rate tests between *D. melanogaster* paralogs and *Anopheles and Rhagoletis STE24*.

Taxon 1	Taxon 2	p-value
Anopheles *STE24* (108)	*D. mel. CG9000* (112)	0.787
Rhagoletis *STE24* (133)	*D. mel. CG9000* (141)	0.629
D. mel. CG9000 (94)	*D. mel. CG9001* (209)	< 0.00001*
D. mel. CG9000 (96)	*D. mel. CG9002* (194)	< 0.00001*
D. mel. CG9000 (100)	*D. mel. CG7573* (234)	< 0.00001*
D. mel. CG9000 (64)	*D. mel. CG30461* (227)	< 0.00001*
D. mel. CG9001 (137)	*D. mel. CG9002* (120)	0.289
D. mel. CG9001 (139)	*D. mel. CG7573* (157)	0.296
D. mel. CG9001 (85)	*D. mel. CG30461* (166)	< 0.00001*
D. mel. CG9002 (116)	*D. mel. CG7573* (155)	0.018
D. mel. CG9002 (87)	*D. mel. CG30461* (192)	< 0.00001*
D. mel. CG7573 (91)	*D. mel. CG30461* (166)	< 0.00001*

* = significant rate differences after Benjamini-Hochberg correction.
Homo sapiens served as the outgroup in all tests. The number of unique nucleotide differences for each taxon is listed in brackets.
