# Supplementary material for: Type-I Prenyl Protease Function Is Required in the Male Germline of Drosophila melanogaster
Source: G3 (Bethesda). 2012 Jun 1;2(6):629–42. doi: 10.1534/g3.112.002188 (PMC3362292; doi:10.1534/g3.112.002188)
Supplement: Supporting Information [file supp_2.6.629_TableS2.pdf]

Table S2 Penetrance and expressivity test

	♂1	♂2	♂3	♂4	♂5	♂6	♂7	♂8	♂9	♂10	♂11	♂12	♂13	♂14	♂15
3KO/3KO	4	2	0	0	3	1	5	16	0	1	3	10	0	0	2
yw/yw	100+	100+	100+	100+	100+	100+	100+	100+	100+	100+	100+	100+	100+	100+	100+

The triple knock-out (3KO) genotype causes a fully penetrant but variably expressive fertility defect in males**.** Fifteen single triple knock-out males were crossed to four virgin homozygous triple knock-out sibs, and the crosses turned every three to five days and scored for adult progeny (the value in each cell of the table refers to the number of progeny). Controls consisted of age-matched wild type (*yw)* individual males crossed to four virgin wild type (*yw)* females and turned every three to five days, and scoring stopped after progeny numbers exceeded 100.
